# The Functional State of *Thermoplasma acidophilum* Pyruvate Kinase Relies on an Extra Carboxyl-Terminal Sequence

**DOI:** 10.3390/ijms26178410

**Published:** 2025-08-29

**Authors:** Leticia Ramírez-Silva, Héctor Riveros-Rosas, Gloria Hernández-Alcántara, José J. García-Trejo, Alicia Vega-Segura, Martin González-Andrade, A. Jessica Díaz-Salazar, Guillermo Salcedo-Barrientos

**Affiliations:** 1 Departamento de Bioquímica, Facultad de Medicina, Universidad Nacional Autónoma de México, Ciudad de México 04510, Mexico; hriveros@unam.mx (H.R.-R.); ghernandez@bq.unam.mx (G.H.-A.); malivega@bq.unam.mx (A.V.-S.); martin@bq.unam.mx (M.G.-A.); gsalcedoba@ciencias.unam.mx (G.S.-B.); 2 Departamento de Biología, Facultad de Química, Universidad Nacional Autónoma de México, Ciudad de México 04510, Mexico; jjgartre@unam.mx; 3 Departamento de Fisicoquímica, Facultad de Química, Universidad Nacional Autónoma de México, Ciudad de México 04510, Mexico; jessicadiazsalazar@gmail.com

**Keywords:** *Thermoplasma acidophilum*, pyruvate kinase, kinetic constants, quaternary structure, extra carboxyl terminal sequence

## Abstract

Phylogenetic studies of the pyruvate kinase family reveal two clusters: the K^+^-dependent and -independent enzymes. *Thermoplasma acidophilum* pyruvate kinase belongs to the latter but possesses the conserved signature of those K^+^-dependent. Recently, we found two distinct ways for these groups to catalyze. It is interesting to elucidate how the *T. acidophilum* enzyme achieves its active conformation. A structural model of this enzyme revealed the presence of an extra C-terminal sequence (ECTS). To understand its role, an enzyme lacking this sequence from *T. acidophilum* was constructed. We then compared the kinetic parameters, far-UV CD spectra, thermal stability, molecular dynamics simulations, and oligomeric states of both the wild-type and truncated enzymes. We found that the truncated enzyme is aggregated and almost inactive, with residual 20% of the total interactions, and it exhibits a soluble fraction of smaller oligomeric states than the wild-type enzyme. These findings suggest that ECTS plays a crucial role in maintaining its active tetrameric state. This sequence is the first reported in an archaeal pyruvate kinase and is also found in other archaea and bacteria. Phylogenetic analysis of ECTS in pyruvate kinases exhibits a sparse distribution that might be explained if ECTS represents an ancient domain prone to loss.

## 1. Introduction

Previous phylogenetic analyses of the pyruvate kinase (PK) family [1,2] indicate that the PK tree is dichotomic. Those PKs that have E117 (numbering according to rabbit muscle pyruvate kinase (RMPK)) and the conserved signature T113/K114/E117/T120 are K^+^-dependent PKs; whereas those PKs that mostly have K117 and the conserved signature L113/Q114/K117/(L, I, V)120 are K^+^-independent PKs. In this latter cluster, a small group of Euryarchaeota (actually Methanobacteriati [3]) with PKs of the genera *Thermoplasma* and *Aciduliprofundum* were identified. An unusual feature is observed in the members of this group; they all have E117 and possess 2 or 3 of the 4 residues of the conserved signature of the K^+^-dependent enzymes, as observed in the pyruvate kinase from *Thermoplasma acidophilum* (WT-*Tha*PK) (L113/K114/E117/T120). This PK was first suggested to be K^+^-dependent even when the authors used an unusually low K^+^ concentration (7.2 mM) in the reaction mixture and omitted to show the activity of the enzyme without the monovalent cation [4] and later, Johnsen et al. (2019) showed that 10 mM K^+^ increased its activity 30% [5]. This slight increase in WT-*Tha*PK activity might be due to a weak ionic strength effect, as reported previously in several K^+^-independent RMPK mutants [6]. However, if WT-*Tha*PK is a K^+^-independent enzyme, with the presence of most of the residues of the conserved signature of the K^+^-dependent PKs, it is interesting to elucidate how it acquires its active conformation. In this regard, we found that two distinct ways have evolved for the catalysis of the pyruvate kinase reaction, one for the K^+^-dependent PKs with E117 and the other for the K^+^-independent PKs with Lys117 [6]. In this case, it is expected that WT-*Tha*PK acquires its active conformation differently, as it does not exhibit the properties of the aforementioned groups. In this study, we started evaluating the kinetic properties of the WT-*Tha*PK and compared them with previously published data [4,5]. We found that this enzyme is K^+^-independent and exhibits no activation by AMP. Its *K_app_* for MgADP is similar to previous reports, but its *K_app_* for PEP is comparable to that in [4] with AMP, although it is 10 and 15-fold lower than those in [4] and [5] without AMP, respectively. If the kinetic experiments of this work were conducted at the same temperature as previous studies, the *V*_max_ of WT-*Tha*PK would be comparable to that of reference [4] and double that of [5]. As in most PKs, WT-*Tha*PK exhibited a rapid equilibrium random order mechanism. Since a crystal structure is not available for this enzyme, we obtained models for the monomer and tetramer using templates from AlphaFold and PDB 3T05 of Methicillin-resistant *Staphylococcus aureus* pyruvate kinase (MRSA PK), respectively. Unexpectedly, it was observed that the structure of WT-*Tha*PK contained an additional long extra C-terminal sequence (ECTS). To our knowledge, this is the first report of this ECTS in a PK from the Archaea domain. This ECTS has been reported before in several PKs [7,8,9,10,11], but its function remains unknown. To understand its role, the *Tha*PK lacking this ECTS was constructed. We found that, in comparison with the WT-*Tha*PK, the truncated enzyme exhibited *K_app_* for PEP and MgADP 48-fold and 7-fold higher, respectively, and the *V*_max_ of the latter was 8% of the former. Far-UV CD spectra were similar with minor differences between both enzymes, whilst DSC showed that truncated-*Tha*PK was aggregated with only 20% of the total interactions of the wild-type enzyme. Molecular dynamics simulations showed that the flexibility of the WT-*Tha*PK increased by 4 Å during the complete simulation, while the truncated enzyme remained unchanged during the same period. The WT enzyme showed high flexibility in the ECTS; in contrast, the truncated enzyme showed no flexibility in any structural region. BN-PAGE showed that the truncated enzyme is mostly aggregated, with a small soluble fraction composed of lower-molecular-weight oligomeric species compared to that of the wild-type enzyme. Therefore, the instability of its quaternary structure indicates that the role of the ECTS is to stabilize its tetrameric state and thus induce the acquisition of its active conformation. Finally, it is worth mentioning that PKs with ECTS are sparsely distributed across different bacterial and archaeal taxa (Bacillati and Pseudomonadati in bacterial kingdom, and Methanobacteriati and Thermoproteati in the archaeal kingdom). Despite having, in some cases, low identity values between their ECTS, those PKs that conserve it exhibit a similar folding in tertiary and quaternary structures. This finding suggests that ECTS might be a very ancient domain present in the ancestral PKs that is prone to loss.

## 2. Results and Discussion

### 2.1. Purification and Oligomeric State of WT-ThaPK

**Figure 1:**
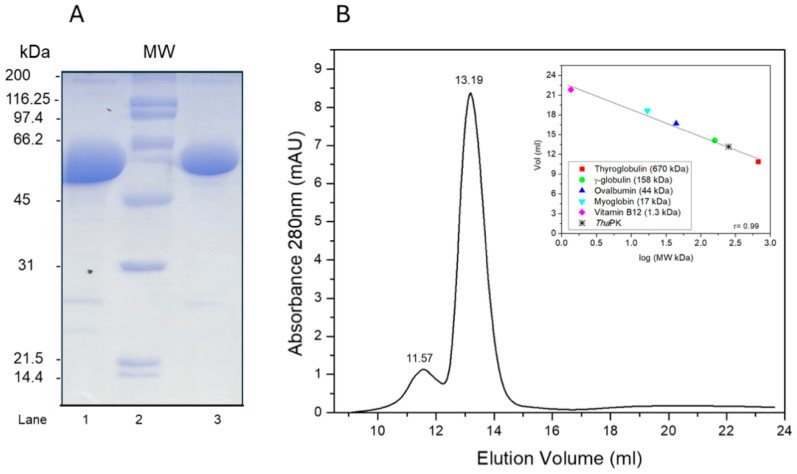
SDS-PAGE at 12% polyacrylamide (**A**) and Gel Filtration column (**B**) of WT-*Tha*PK. In (**A**), the gel was prepared according to [12]. Lanes 1 and 3 were loaded with 20 and 10 µg of protein, respectively. Lane 2 was loaded with the SDS-PAGE Molecular Weight Standards Broad Range of BIORAD. In (**B**), a column Superdex 200 (10/300) from GE Healthcare, previously equilibrated with 50 mM HEPES-(CH_3_)_4_NOH, pH 7.5, 150 mM NaCl, was loaded with Gel Filtration standards from BIORAD indicated in the calibration curve of the inset, then washed and equilibrated once more to load 500 µg of WT-*Tha*PK. The elution volumes of an aggregate of the *Tha*PK and the tetrameric states of the enzyme are indicated as 11.57 and 13.19 mL, respectively.

*WT-Tha*PK was purified as described in the Section 3. The enzyme was 90% pure, as indicated by 12% SDS-PAGE (Figure 1A). Since thrombin was unable to cleave the His_6_ tag, the experiments were conducted with the 20 additional residues. Therefore, the monomer molecular weight was 61,340.84 Da (59,147.48 of monomer plus 2193.26 of the 20 amino acid residues of the His_6_ tag and thrombin cleavage site). Coincidentally, after SDS-PAGE of these PK samples according to [12], the linear regression analysis of the Rf for the bands of the molecular weight standards (lane 2) with the software Alpha-Digidoc^TM^ 1000 software (AlphaEaseFCTM from Alpha Innotech Corp., San Leandro, CA, USA) calculated a molecular weight of 61 kDa for the monomer of WT-*Tha*PK. It is worth noting that we were unable to remove the His_6_ tag and protease cleavage site in any of the previously studied PKs in our lab [2,13]. However, in those studies, we obtained a small quantity of PK without the His_6_ tag and demonstrated that the kinetic behavior of the enzymes was similar to that with the His_6_ tag. In this case, no WT-*Tha*PK free of His_6_ tag and thrombin cleavage site was obtained after incubation for 24 h with the Thrombin Clean Cleave kit from SIGMA (SDS-PAGE 12%). A gel filtration column was used to determine the oligomeric state of WT-*Tha*PK (Figure 1B). The calibration curve was obtained using the Gel filtration standards shown in the inset. A first peak, with an elution volume of 11.57 mL, exhibited less than 10% of the PK activity of the second peak, with an elution volume of 13.19 mL. The molecular weights of the first and the second peaks were 629.37 and 252.63 kDa, respectively. This result indicates that we have a small fraction of *Tha*PK aggregated and the expected tetramer (245.36 kDa).

### 2.2. pH Profile of WT-ThaPK

To determine the optimum pH for WT-*Tha*PK, the activities of the enzyme preparations were measured at different pH values. A saturation curve for PEP was obtained at each pH to make sure that the activities of the pH profile were in *V*_max_ conditions. Therefore, the activities of WT-*Tha*PK at pH values 5.5, 6, 6.12, 6.5, 6.85, 7.14, and 7.53 were determined in the presence of 3 mM PEP; whereas those for pH values 8.06, 8.54, and 9 were determined with 5 mM PEP. As shown in Figure S1, the optimum pH for the activity of WT-*Tha*PK was 6.5. In contrast, two previous studies of this PK reported 7.5 as the optimum pH; however, they do not present the results or indicate the experimental conditions of the pH assay [4,5].

### 2.3. Effect of Monovalent Cations and Allosteric Effectors on WT-ThaPK

To explore whether monovalent cations activated WT-*Tha*PK, the effect of Li^+^, Na^+^, K^+^, NH_4_^+^, Rb^+^, and Cs^+^ was investigated. As shown in Figure S2A, no activation of the enzyme was observed with 10 to 100 mM of monovalent cations, nor with 1 to 10 mM of K^+^. In contrast, a previous study suggested that WT-*Tha*PK is K^+^-dependent. However, the authors used an unusually low K^+^ concentration (7.2 mM) in the reaction mixture and did not demonstrate the enzyme’s activity in the absence of K^+^ [4]. Moreover, if WT-*Tha*PK was a K^+^-dependent enzyme, it should have less affinity for K^+^ than RMPK due to the presence of the substitution of T113 for L113 (RMPK numbering) present in its signature as described in [14]. The optimum K^+^ concentration for activation of the PK reaction is 100 mM [15], and the activating effect of K^+^ is 10,000-fold [15,16]. In another study, Johnsen et al. (2019) showed that 10 mM K^+^ increased its activity by 30% [5]. This slight increase in the activity of WT-*Tha*PK might be due to an ionic strength effect as reported previously in several K^+^-independent RMPK mutants [6]. Therefore, taken together, our present results reported here clearly show that WT-*Tha*PK is a K^+^-independent PK.

To achieve maximal activity of WT-*Tha*PK, the effects of 3PG, Rib-5P, Glc-6P, AMP, and Fru 1,6 BP were tested in the presence of the Km for ionized PEP and saturating concentrations of MgADP and free Mg^2+^. As shown in Figure S2B, there was no effect of either of the effectors. It was previously reported that WT-*Tha*PK was activated by AMP [4]; however, neither in reference [5] nor did we find any effect of the nucleotide.

### 2.4. Kinetic Parameters of WT-ThaPK in the Presence of Mg^2+^ and Mn^2+^

**Figure 2:**
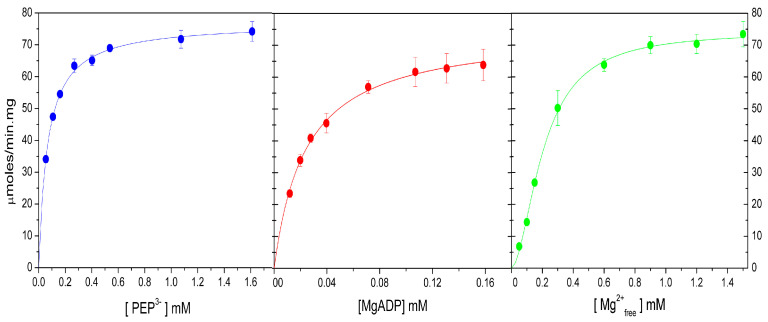
Saturation curves for PEP^3-^, MgADP, and free Mg^2+^ of *Tha*PK. PEP^3-^ and MgADP kinetics were performed under saturation concentrations of the other substrate. The concentrations of PEP varied from 0.57 to 1.6 mM at a fixed 0.24 mM MgADP, and those of MgADP were varied from 0.012 to 0.16 mM at a fixed 0.66 mM PEP. The concentrations of free Mg^2+^ for the PEP^3-^ and MgADP saturation curves were 1.41 mM. For the saturation curves of free Mg^2+^, the concentrations of the divalent cation were varied from 0.05 to 1.5 mM at fixed concentrations of 0.24 mM MgADP and 0.66 mM PEP^3−^. The reaction mixtures contained 50 mM MOPS-(CH_3_)_4_NOH, pH 6.5, 0.2 mM NADH, 10 μg/mL LDH, and the ionic strength was maintained constant at 200 mM with (CH_3_)_4_N^+^. The means and standard deviations from three different experiments are shown. The experiments were conducted at 45 °C.

PK has an absolute requirement for divalent cations because they are essential for phosphate transfer [17]. Therefore, the effects of Mg^2+^, the physiologically divalent cation, and of the “ancient Mn^2+”^ [18] on the activity of WT-*Tha*PK were explored. The experiments were performed in the absence of AMP and the presence of 0.2 M constant ionic strength with (CH_3_)_4_N^+^. Saturation curves for PEP^3-^, MgADP, and free Mg^2+^ are shown in Figure 2, and the apparent kinetic constants of these data are listed in Table 1. As observed in Figure 2, WT-*Tha*PK exhibited hyperbolic saturating curves for PEP^3-^ and MgADP. This enzyme showed a similar *K_app_* for PEP^3-^ and free Mg^2+^ and a 10-fold lower *K_app_* for MgADP than RMPK, a constitutively active PK. In comparison with previous kinetic reports of WT-*Tha*PK without AMP, the *K_app_* for PEP^3-^ was 10 and 15-fold lower than that in references [4] and [5], respectively, and similar to that with AMP in reference [4]; whereas the *K_app_* for MgADP was similar. Initially, Kinetic assays of WT-*Tha*PK were performed at 25 °C and 45 °C in the presence of identical reaction mixtures; the specific activities were 20 and 75 μmol/min·mg, respectively. The temperature coefficient (Q_10_) (https://www.physiologyweb.com, accessed on 26 March 2025) was 1.94; i.e., the rate almost doubled every 10-degree increase. In this regard, as indicated in Table 1, the *V*_max_ of 75 μmol/min·mg at 45 °C would rise to 202 μmol/min·mg at 60 °C (the temperature at which previous studies were performed), this activity would be similar to that described in reference [4], but 2-fold higher than that in reference [5].

**Figure 3:**
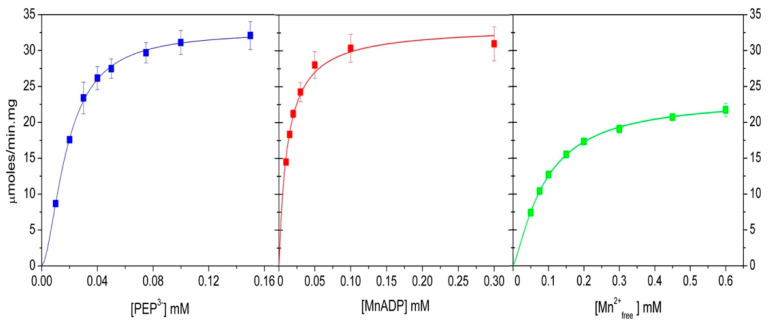
Saturation curves for PEP^3-^, MnADP, and free Mn^2+^ of *Tha*PK. PEP^3−^ and MnADP kinetics were performed under saturation concentrations of the other substrate. The concentrations of PEP^3-^ were varied from 0.01 to 0.15 mM at a fixed 0.1 mM MnADP, and those of MnADP were varied from 0.01 to 0.3 mM at a fixed 0.34 mM PEP^3−^. The concentrations of free Mn^2+^ for the PEP^3−^ and Mn-ADP saturation curves were 0.85 mM. For the saturation curves of free Mn^2+^, the concentrations of free divalent cation were varied from 0.05 to 0.6 mM at a fixed concentration 0.1 mM Mn-ADP and 0.34 mM PEP^3−^. The other experimental conditions were the same as those in Figure 2. The means and standard deviations from three different experiments are shown. The experiments were conducted out at 45 °C.

Figure 3 and Table 2 display the saturation curves for PEP^3-^, MnADP, and free Mn^2+^, along with their corresponding apparent kinetic constants, respectively. Despite *V*_max_ with Mg^2+^ being ~2 to 3-fold higher than with Mn^2+^, the *K_app_* for the substrates were 2 to 4-fold lower with Mn^2+^ than with Mg^2+^; therefore, the catalytic efficiencies of WT-*Tha*PK with Mg^2+^ and Mn^2+^ were alike. In comparison with the Crenarchaeota (currently Thermoproteati [3]), *Thermofilum pendens* pyruvate kinase (*Tp*PK) that exhibits one of the smallest *K_app_* for Mn^2+^ [2], WT-*Tha*PK showed a *K_app_* for the divalent cation 4.5-fold higher, but its *K_app_* for PEP^3-^ and MnADP were 56 and 20-fold lower, respectively, than those for *Tp*PK.

These results indicate that although WT-*Tha*PK did not exhibit high activity, it is kinetically very efficient due to the high affinities for its substrates.

### 2.5. Bi-Substrate Kinetics of WT-ThaPK

**Figure 4:**
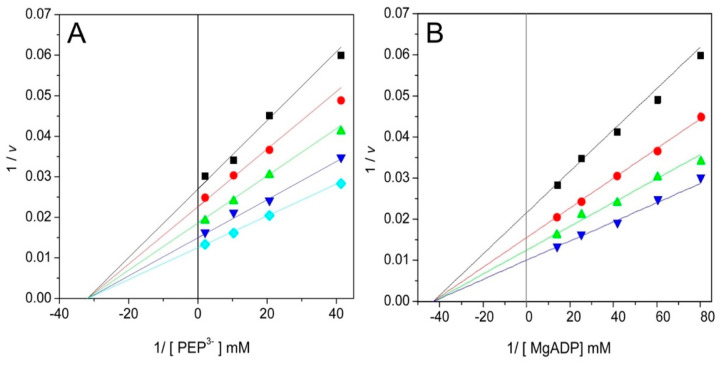
Double reciprocal plots from the initial velocity data of the reaction catalyzed by *Tha*PK. The reciprocals of the PEP^3-^ and MgADP complexes’ concentrations are shown in each graph’s abscissae. The variable fixed concentrations of MgADP in plot (**A**) were 0.0125 (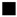), 0.0166 (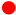), 0.024 (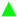), 0.40 (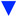), and 0.071 mM (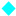). The variable fixed concentrations of PEP^3-^ in plot (B) were 0.024 (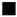), 0.048 (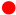), 0.097 (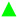), and 0.48 mM (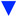). In both plots, the Mg^2+^_free_ concentration was kept constant at 1.41 mM. The other experimental conditions were as shown in Figure 2. The addition of *Tha*PK initiated the reaction; the enzyme amounts ranged from 0.15 to 0.3 μg/mL. The fitted data are shown in Table 3.

Bi-substrate kinetics of PK were first performed in the K^+^-dependent, well-known RMPK. Boyer’s group [19] demonstrated that this enzyme follows a random-order rapid equilibrium kinetic mechanism at saturating concentrations of K^+^, a finding later confirmed by others [17,20,21]. However, when RMPK is in the absence of K^+^, the mechanism changes to an ordered one with PEP as the first substrate [21]. In contrast, without K^+^, the K^+^-independent mutant E117K-RMPK exhibits the same random-order rapid equilibrium kinetic mechanism [21]. This finding is quite general for all PKs; they follow this kinetic mechanism, either K^+^-dependent PKs with saturating concentrations of K^+^ (PK of *Vibrio cholerae* I [13]) or K^+^-independent PKs without K^+^ (*Tp*PK [2] and PK of *Vibrio cholerae* II [13]). In this context, bi-substrate kinetics of WT-*Tha*PK were carried out in the absence of K^+^ and the presence of Mg^2+^ as the divalent cation (Figure 4). These experiments were conducted at various concentrations of one substrate and at fixed concentrations of the other. The double reciprocal plots of the initial velocities versus the ionized PEP concentrations intersected on the 1/S axis and to the left of the 1/*v* axis (Figure 4A). When the concentration of MgADP was varied, the lines intersected on the 1/S axis and to the left on the 1/*v* axis (Figure 4B). These results indicate either an ordered steady state or a rapid equilibrium random-order kinetic mechanism. These data were globally fitted to the equation described in Table 3, and the kinetic constants obtained are listed. It is relevant to mention that K_m_ for MgADP is one of the smallest reported so far for a pyruvate kinase.

### 2.6. Dead-End Inhibition Studies of WT-ThaPK

**Figure 5:**
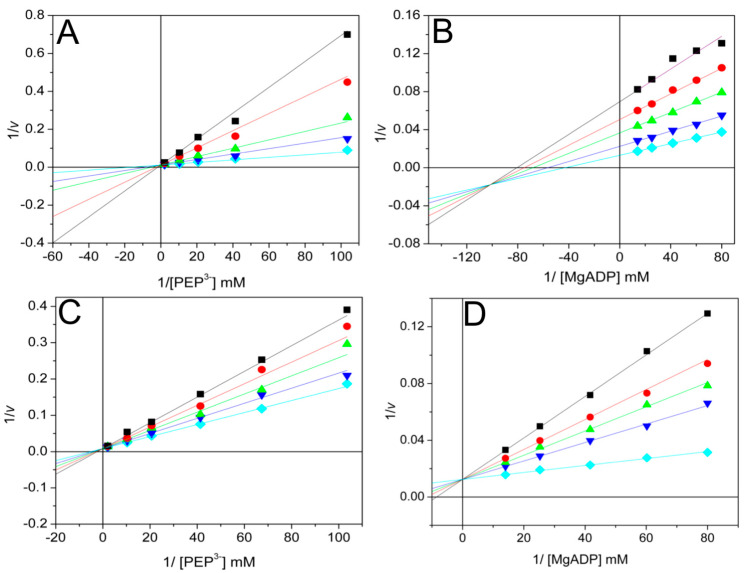
Dead-end inhibition patterns for oxalate versus PEP^3−^ (**A**) and MgADP (**B**), and AMP versus PEP^3-^ (**C**) and MgADP (**D**). The experimental conditions are indicated in Figure 4. The reciprocals of ionized PEP and MgADP concentrations are indicated on the abscissas of each graph. In plot A, the variable concentrations of PEP^3-^ were 0.0097, 0.024, 0.048, 0.097, and 0.484 mM. The fixed variable concentrations of oxalate were 0 (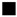), 0.05 (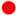), 0.1 (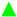), 0.25 (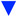), and 0.4 mM (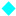). The fixed concentrations of free Mg^2+^ and MgADP were 1.88 and 0.257 mM, respectively. In plot B, the variable concentrations of MgADP were 0.0125, 0.0166, 0.024, 0.0396, and 0.0713 mM. The fixed variable concentrations of oxalate were 0 (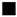), 20 (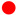), 30 (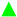), 40 (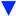), and 60 (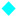). The fixed concentrations of free Mg^2+^ and total PEP were 1.88 and 1.127 mM, respectively. The reaction was initiated by the addition of *Tha*PK; the enzyme amounts of enzyme ranged from 0.15 to 0.3 μg/mL.

The use of dead-end inhibitors provides a valuable tool to probe the kinetic mechanisms of enzymes [22]. In this work, oxalate and AMP were used as dead-end analogs of PEP [23] and ADP [2], respectively. The patterns of oxalate inhibition versus ionized PEP and MgADP were competitive (Figure 5A) and mixed (Figure 5B), respectively. With AMP, the inhibition was competitive either with ionized PEP (Figure 5C) or MgADP (Figure 5D). The data were globally fitted to the equations that describe linear competitive inhibition or linear mixed inhibition. The inhibition patterns and inhibition constants are shown in Table 4. The data indicate that oxalate acts as a competitive inhibitor of PEP, and AMP functions as a competitive inhibitor of MgADP. This indicates that the analogs and the substrates bind to the same site. Unexpectedly, AMP and PEP appear to occupy the same site as well. In contrast, the inhibition pattern of AMP versus PEP for *Tp*PK was mixed [2]. To elucidate the competitive inhibition pattern of AMP versus PEP in Figure 5C, a molecular docking of WT-*Tha*PK was performed as described in the Section 3. As shown in Figure S3, AMP and PEP bind to the active site of the enzyme. Upon close examination, it is observed that the skeleton of AMP overlaps with that of PEP, which explains the competitive behavior between them. On the other hand, the mixed-type inhibition with α < 1 (factor affecting Ki) for oxalate versus MgADP indicates that oxalate forms a non-productive ternary complex and thereby diminishes the *V*_max_, where the enzyme-oxalate binary complex has a higher affinity for MgADP than the free enzyme. Therefore, the results obtained with dead-end inhibitors indicate that WT-*Tha*PK follows a rapid-equilibrium random-order kinetic mechanism, as reported previously for other PKs [2,13,20].

### 2.7. The Structural Model of the WT-ThaPK

**Figure 6:**
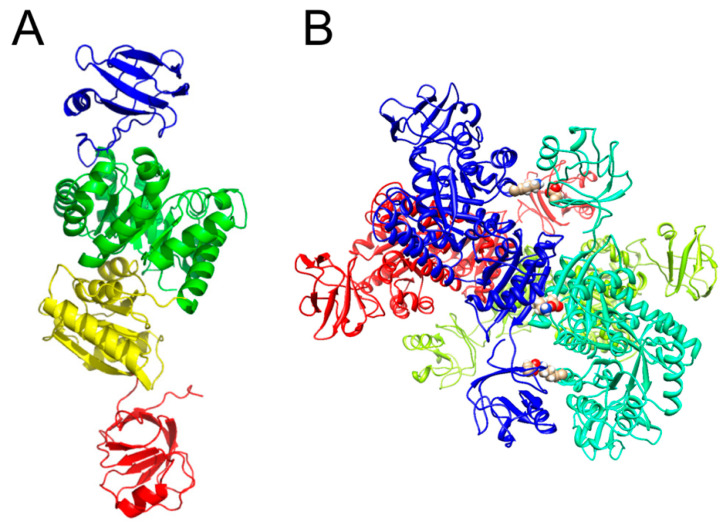
Structural model of the monomer (**A**) and the tetramer (**B**) of the *Tha*PK. The models of the monomer and the tetramer were built using as a template the model of AlphaFold AF-P32044-F1 model as a template and the PDB 3T05 of MRSA PK, respectively. Both models were performed with the online software SWISS-MODEL (https://swissmodel.expasy.org/, accessed on 26 March 2025) [22]. The monomer (**A**) was colored by domains: A (green), B (blue), C (yellow), and ECTS (red). The tetramer (**B**) showed three salt bridges between the εN of K217 of domain A (chain B (cyan)) and COO^−^ of D462 of ECTS (chain A (blue)), between COO^−^ of D462 of ECTS (chain B (cyan)) and εN of K217 of domain A (chain A (blue)) and between COO^−^ of D444 of domain C (chain B (cyan)) and εN of K446 of domain C (chain A (blue)). The same interactions were found between chains D (red) and C (green). These salt bridges were found with the online version of the software PLIP (Protein Ligand Interaction Profiler) (https://doi.org/10.1093/NAR/GKAB294) (accessed on 26 March 2025) [29].

The models of the monomer and the tetramer of WT-*Tha*PK were built using the AlphaFold AF_P32044-F1 model as a template and the PDB 3T05 of MRSA PK as a template, respectively. Both models were constructed with the online version of the software SWISS-MODEL (https://swissmodel.expasy.org/, accessed on 21 March 2025) [24]. The monomer model revealed that this PK exhibited an ECTS (Figure 6A). To our knowledge, this is the first report of an ECTS in archaea. In contrast, a similar structural domain was first reported in the PK from *Geobacillus stearothermophilus* (*Gst*PK) [7], followed by the PKs of *Bacillus psychrophilus* and *Bacillus licheniformes* [8], of *Bacillus subtilis* [9], of *Listeria delbrueckii* and cyanobacteria *Synechocystis* sp. [10], and of MRSA PK [11]. The ECTS of the *Gst*PK has approximately 110 amino acid residues. A part of the sequence is highly homologous to the phosphoenolpyruvate: sugar phosphotransferase system, pyruvate phosphate dikinase, and phosphoenolpyruvate synthase enzyme I [7,25]. It includes a PEP binding motif highly conserved around a His, which is phosphorylated during the enzymatic reaction [25]. However, its role in PKs remains unclear [11,26]. To model the tetrameric structure of WT-*Tha*PK, the biological assembly of PDB 2E28 of *Gst*PK [27] and that of PDB 3T05 of MRSA PK [28] were used. The PDB 3T05 was more suitable for modeling WT-*Tha*PK. The analysis of the tetrameric structure of the WT-*Tha*PK model revealed the presence of three salt bridges between dimers (Figure 6B). Two of these were also observed in PDB 3T05; the new one is the one formed between the domains C of contiguous chains. These salt bridges were found with the online version of the software PLIP (Protein-Ligand Interaction Profiler) (https://doi.org/10.1093/NAR/GKAB294) (accessed on 26 March 2025) [29]. After the models were obtained, their quality was evaluated with Molprobity.

### 2.8. Kinetic Parameters for ECTS Truncated-ThaPK

The AlphaFold3 model of WT-*Tha*PK (Figure 6) revealed that the enzyme exhibits an ECTS, as previously reported in several PKs from *Bacillus* and other bacteria [7,8,9,10,11]. This domain was deleted from *Gst*PK [26] and from MRSA PK [11] to understand its function. In the absence of their ECTS, both PKs conserved their allosteric effect by AMP; their kinetic constants and oligomeric states were similar to those found in their wild-type enzymes. The truncated bacillus PK was 4 °C less thermostable than the wild-type enzyme. In the wild-type enzymes, no hydrolysis of PEP was observed in the absence of MgADP; therefore, aside from a slight thermostabilization, the available data leave the function of this ECTS as unknown. Therefore, to study the role of the extra C-terminal sequence of WT-*Tha*PK, the truncated enzyme was constructed as described in the Section 3. Table 5 presents the kinetic parameters for the truncated-*Tha*PK. In comparison with WT-*Tha*PK, the truncated enzyme exhibited sigmoidal kinetics instead of a hyperbolic one. The truncated enzyme displayed about 8% of the *V*_max_ of the WT-*Tha*PK, and the *K_0.5_* for ionized PEP and MgADP were 48 and 7-fold higher, respectively, than those of the wild-type enzyme. These results suggest that either the truncated enzyme is essentially inactive, or approximately 8% of the total enzyme is active, while the remaining protein is inactive and denatured. However, even if 8% of the enzyme is active, the binding site of the substrates has been severely modified.

### 2.9. Circular Dichroism Spectra and Differential Scanning Calorimetry of WT-ThaPK and ECTS Truncated-ThaPK

**Figure 7:**
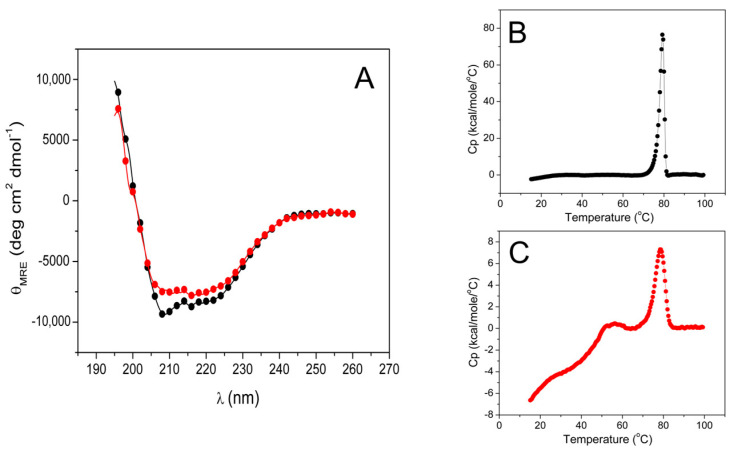
Far UV CD spectra (**A**) and Differential scanning calorimetry (**B**) of WT-*Tha*PK (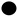) and (**C**) of ECTS truncated-*Tha*PK (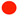). In (**A**), the spectra were obtained in mixtures containing 100 μg/mL of the WT-*Tha*PK or truncated-*Tha*PK in 20 mM NaH_2_PO_4-_NaOH, pH 7.0, at 25 °C in a 0.1 cm cell pathway. CD is expressed as θ_MRE_ (molar residue ellipticity). In (**B**,**C**), the protein concentrations were 1.0 mg/mL, 16.3 μM monomer of the wild type, and 19.41 μM monomer of the ECTS-truncated enzyme. The enzymes were diluted in 50 mM MOPS-(CH_3_)_4_NOH, pH 6.5, containing 0.3 mM oxalate, 0.1 mM ATP, and 1.05 mM Mg^2+^. The scan rate was 1.5 °C/min. The T_ms_ were 79.2 and 78.4 °C for WT-*Tha*PK and truncated-*Tha*PK, respectively. ∆H (cal/mol °C) were 230,946.6 and 46,845.8 for the WT-*Tha*PK and truncated-*Tha*PK, respectively.

Circular Dichroism (CD) is an ideal technique for estimating the structural integrity of recombinant, purified proteins, either with one or several modifications in their amino acid sequence, or for evaluating the impact of mutations on their conformation or stability [30]. Pursuing this goal, the far UV-CD spectra of WT-*Tha*PK and truncated-*Tha*PK were performed (Figure 7A). The spectra showed minor differences between the WT and the truncated-*Tha*PK as molar residue ellipticity. To explore the differences in thermal stability between the two forms of the enzyme, differential scanning calorimetry (DSC) was performed. The experiments were conducted in the absence and presence of ligands (Mg^2+^, oxalate, and ATP), and it was found that the *T_ms_* for each enzyme were similar in both conditions, with a small gain in enthalpy (5–8%) upon addition of the ligands. Figure 7B,C show the endotherms in the presence of ligands of WT-*Tha*PK and of the truncated-*Tha*PK, respectively.

The WT enzyme exhibits a classical thermal unfolding profile, while the truncated enzyme displays a distinct unfolding pattern. Interestingly, the truncated enzyme shows an unfolding trace opposite to that observed for methanodextrin glucosidase, a protein known to undergo unfolding before aggregation [31]. Drawing from the behavior described in [31], it is inferred that, in the case of the truncated *Tha*PK, heating from 15 to 60 °C causes disaggregation of protein aggregates. This is followed by the unfolding of the remaining folded fraction, which exhibits a melting temperature (Tm) of 78.4 °C, comparable to that of the WT enzyme (79.2 °C). Notably, the unfolding enthalpy of the truncated enzyme is only 20% of that measured for the WT enzyme. This significant reduction suggests a substantial loss of native structural interactions in the truncated form. The observed loss is most likely due to aggregation, implying that the endothermic unfolding transition corresponds to only a minor population of properly folded protein.

### 2.10. Molecular Dynamics Simulations of WT-ThaPK and ECTS Truncated-ThaPK

**Figure 8:**
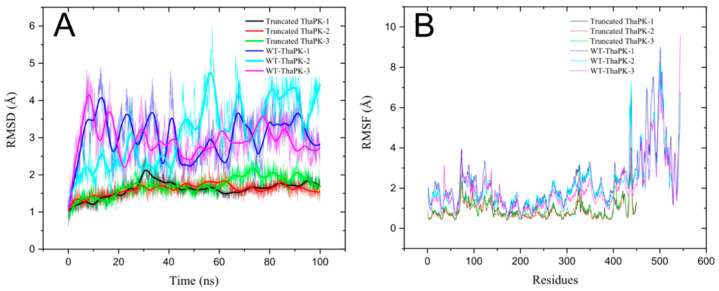
RMSD and RMSF from molecular trajectories of ECTS-truncated *Tha*PK and WT-*Tha*PK (**A**,**B**). Triplicates of molecular dynamics in the presence of Mg^2+^, K^+^, oxalate, and ATP were carried out, and the analyses were made with CPPTRAJ [32].

The RMSD is used to estimate the global flexibility of a protein’s structure. The analysis considers the mean amount of movement of the backbone atoms around the complete protein. RMSF evaluates the localized flexibility of one or several amino acid residues throughout the whole structure. Figure 8A shows three replicates of the RMSD of WT-*Tha*PK and truncated-*Tha*PK. The flexibility of the wild-type enzyme increased ~4Å with fluctuations of 2 to 4 Å during the 100 ns, whereas the truncated enzyme remained unchanged throughout the whole simulation. This means that the truncated enzyme has reduced its overall conformational flexibility and likely impacts its function. Figure 8B shows three replicates of the RMSF of the wild-type enzyme and the truncated enzyme. The wild-type enzyme shows high flexibility in the extra C-terminal sequence (up to ~8 Å) and a minor flexibility (less than 4 Å) in the region of the B domain (residues 65 to 156). In contrast, no structural region showed flexibility in the enzyme lacking the ECTS. This result suggests that this ECTS may be involved in the flexibility required for the enzyme to acquire its active conformation.

### 2.11. D Blue Native Gel Electrophoresis and 2D SDS-PAGE of WT-ThaPK and ECTS-Truncated ThaPK

**Figure 9:**
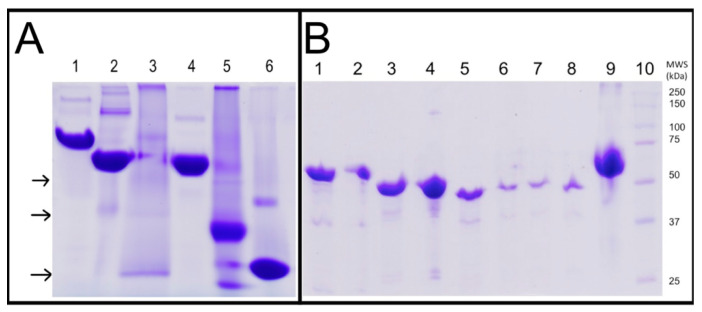
One-dimensional Blue Native Gel Electrophoresis (BN-PAGE) (**A**) and Two-dimensional SDS-PAGE of WT-*Tha*PK and ECTS truncated-*Tha*PK (**B**). (**A**) 1D BN-PAGE was carried out as described in Section 3.10 of the isolated pyruvate kinases (lanes 1–3) and Molecular Weight Standards (MWS, lanes 4–6). The lanes were loaded with 50 µg/well as follows: (1) WT-*Tha*PK (MW = 245.3 kDa); (2) *Tp*PK (MW = 215.9 kDa); (3) truncated-*Tha*PK (MW = 206.0 kDa); (4) Catalase (MW = 232.0 kDa); (5) Acylase (MW = 86.0 kDa); (6) Bovine Serum Albumin (BSA) (MW = 66.0 kDa). In lane 3 loaded with the truncated-*Tha*PK, most of the protein aggregated at the top of the gel, and three protein bands (indicated by black arrows) appeared as apparent smaller aggregation states of the enzyme. These smaller bands were resolved in 2D-SDS-PAGE. (**B**) 2D-SDS-PAGE was carried out in parallel with 1D-BN-PAGE, using normally loaded samples of the same PKs as those resolved in BN-PAGE. Lanes 1 (WT-*Tha*PK), 3 (*Tp*PK), 5 (truncated-*Tha*PK), 9 (BSA), and 10 (MW Standards) were loaded normally with 10 µg/well. Lanes 2, 4 and 6–8 were loaded with the major protein bands excised from a non-stained version of the 1D-BN-PAGE of panel (**A**), corresponding to WT-*Tha*PK (major band of lane 1 in panel (**A**), loaded into lane 2 of panel (**B**)); *Tp*PK (major band of 2 in panel (**A**), loaded into lane 4 of panel (**B**)); truncated-*Tha*PK (lane 3 in panel (**A**), upper arrow band (174.1 kDa) loaded into lane 6 in panel (**B**); middle arrow band (117.0 kDa) loaded into lane 7 in panel (**B**); lower arrow band (53.7 kDa) loaded into lane 8 in panel (**B**)).

To verify the oligomeric state of the truncated enzyme, a BN-PAGE was run (Figure 9A). Lane 3 was loaded with the truncated-*Tha*PK; most of the protein aggregated at the well of the lane, and three protein bands (indicated by black arrows) appeared at apparent smaller association states than the tetrameric native state of the enzyme. These smaller bands were resolved in 2D-SDS-PAGE (Figure 9B) and confirmed the identity as smaller aggregation states of the truncated-*Tha*PK. A linear regression analysis of the Rf of these bands carried out using the molecular weights of the standards indicated (lanes 1–2 and 4–6), with the online version software Alpha-Digidoc™ showed that the molecular weights of these smaller bands (upper to lower black arrows) correspond to 174.1 ± 2.7 kDa, 117.04 ± 4.9, and 53.7 ± 5.4 kDa. These estimations were derived from independent triplicate BN-PAGE gels and their linear regression analysis with Alpha-Digidoc™ (including Average and Standard Deviation (STD) values). The major band (lowest arrow) corresponds to the monomer of truncated-*Tha*PK (MW 51.51 kDa). The other two bands might correspond to dimeric (≈117.04) and trimeric (≈174.13 kDa) forms of the truncated-*Tha*PK. All these three bands (lanes 6–8) migrated essentially in the same position as the normally loaded truncated-*Tha*PK (lane 5), co-migrating with the MWS of 50 kDa, as expected from the MW of the ECTS truncated-*Tha*PK monomer (51.51 kDa), thus proving their identity as different aggregation states of the same truncated-*Tha*PK. This result indicates that the ECTS in WT-*Tha*PK is essential for the stability of the tetrameric state, contrary to *Gst*PK [26] and MRSA [11], which conserve their tetrameric state in the absence of the ECTS. It is relevant to notice that the BN-PAGE did not show a band corresponding to the MW of the truncated-tetrameric enzyme; therefore, the residual activity (8%) found in this enzyme might be due to the different oligomeric forms. In summary, the almost inactive truncated enzyme, the loss of native interactions, and the instability of the tetrameric state indicate that the role of the extra carboxyl-terminal sequence is to stabilize its quaternary structure and facilitate the acquisition of its active conformation.

Molecular exclusion chromatography, as that performed for WT-*Tha*PK in Figure 1B, could not be used to determine the oligomeric state of the truncated enzyme because it could not be isolated in sufficient quantities due to aggregation.

### 2.12. Is the Extra Carboxyl-Terminal Sequence (ECTS) Widely Distributed Along the PKs?

**Figure 10:**
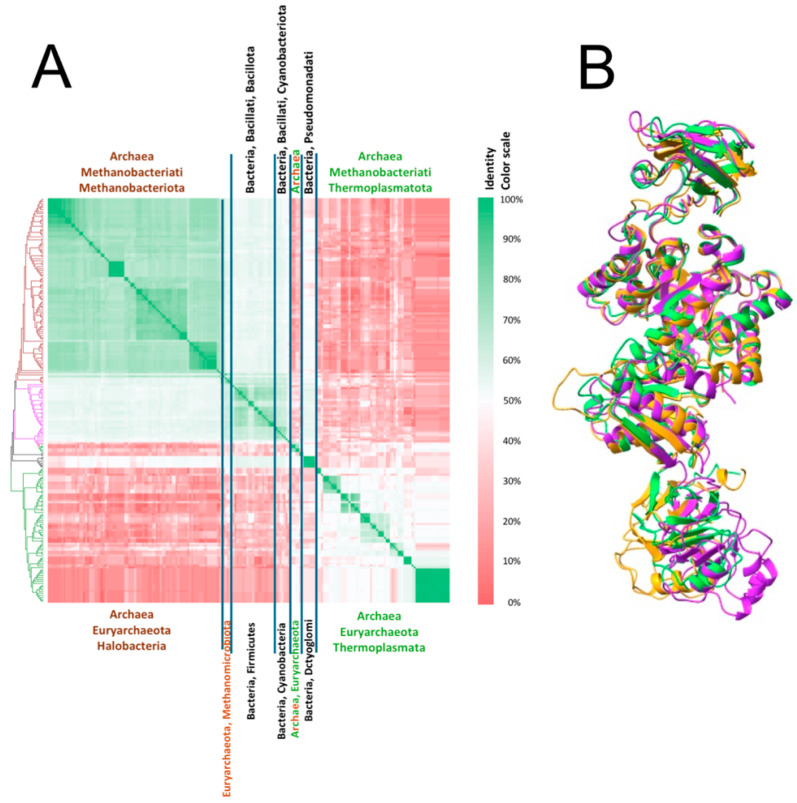
Matrix of the identity of the amino acid sequences of the ECTS of pyruvate kinases retrieved from a Blast (**A**) and Structural alignment in 3D of the PKs from *Halorubrum terrestre* (orange), *Geobacillus stearothermop*hilus (magenta), and *Thermoplasma acidophilum* (green) (**B**). In (**A**), the matrix was constructed with 200 sequences of PKs that had ECTS. A phylogenetic analysis of these sequences is shown in the left portion of the matrix. Of the 200 sequences, 163 were from archaea (68 thermoplasmatota and 95 methanobacteriota). From the latter, 90 were halobacteria, 4 methanomicrobiota, and 1 methanobacteriota unidentified. In regard to bacteria, there were 37 sequences, 20 from bacillus (firmicutes), 8 cyanobacteria, 2 thermodesulfobacteriota, and 7 dictyoglomota. The identity between the amino acid sequences is indicated by the color scale (right side of the matrix). In (**B**), three PKs from the major taxonomic groups having ECTS were structurally aligned: *Halorubrum terrestre* PK (halobacteria (orange)), *Gst*PK (bacillus (magenta)), and *Tha*PK (thermoplasmata (green)).

As described in the Section 3, after BlastP searches, 200 non-identical ECTS sequences were retrieved, and all of them belong to PKs with an extra C-terminal domain (after A, B, and C domains). A phylogenetic tree was constructed using these 200 sequences of PK, which included the additional C-terminal sequence. The phylogenetic tree (left side of Figure 10A) indicates that the ECTS was found only in Archaea (163 sequences) and Bacteria (37 sequences) domains (we did not find ECTS in Eukarya). In Archaea, we found 95 Methanobacteriota (mostly Halobacteria) and 68 Thermoplasmatota (*Tha*PK belongs to this last phylum). In Bacteria, we found 20 Bacillus (Firmicutes) (*Gst*PK and MRSA PK belong to this phylum), 8 Cyanobacteria, 2 Thermodesulfobacteriota, and 7 Dictyoglomata. However, we need to point out that the majority of Archaea and Bacteria possess a PK without an ECTS. Besides the distribution of the PKs containing ECTS, Figure 10A shows the identity of the amino acid sequences between the ECTS. As observed with the color scale identity, the identity between the sequences of Methanobacteria (Halobacteria) is >60%; between the Bacilli is >50%; and between the Thermoplasmatota is >90% and <40% with the closest and farthest neighbors, respectively. On the other hand, the identity between phylogenetic groups, i.e., between Methanobacteriota and Bacillota, is <50%, and between these two groups and Thermoplasmatota is <10%. These results indicate that the ECTS phyletic distribution is limited and can be found only in some taxa. On the other hand, the domain containing ECTS is very tolerant to amino acid substitutions, as can be observed in the matrix of identity values. Indeed, the sequences of those of Thermoplasmatota seem not to be closely related to the extra C-terminal sequence found in other groups.

### 2.13. Structural Alignment in 3D of PKs That Possess ECTS and Belong to Different Taxonomic Groups

To elucidate if a similar folding pattern was observed in the ECTS of the PKs of the different taxonomic groups found in the phylogenetic tree of PKs that contain this domain (left side of Figure 10A), a structural alignment of one PK from each group (methanobacteriota, bacillota, and thermoplasmatota) is shown in Figure 10B. It is observed that A, B, and C domains of PKs are well-preserved and overlap, whereas their ECTS structures do not overlap but contain similar secondary structures, specifically a three-layer β/β/α architecture. This finding agrees with the proposal that PKs containing the ECTS are more tolerant to amino acid substitutions in comparison to the A, B, and C domains of PK.

### 2.14. Are the Pyruvate Kinases That Possess an Extra C-Terminal Sequence (ECTS) Clustered Together in a Global Phylogenetic Tree?

**Figure 11:**
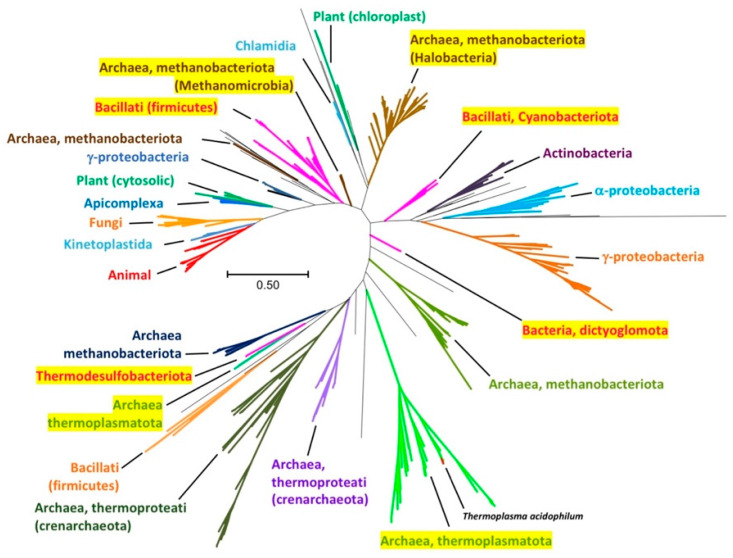
Global tree of the family of PK, containing or not the ECTS. Those taxonomic clusters that have this ECTS are highlighted in yellow, and the sequence of *Tha*PK is indicated in the green branch of Archaea, specifically Thermoplasmatota. The phylogeny was inferred using the Maximum Likelihood method and the Le-Gascuel model of amino acid substitutions. The tree with the highest log likelihood is shown. The evolutionary rate differences among sites were modeled using a discrete Gamma distribution across 5 categories (+G, with a parameter of 0.6761). Evolutionary analyses were conducted in MEGA12.

To answer this question, a sample of 426 representative sequences of PK, reported previously [2], was included in our analyses. The 200 PK sequences that contained an ECTS were added to the first 426 representative PK sequences. After duplicated sequences were deleted, a total of 496 PK full-length sequences were used to build a global phylogenetic tree (Figure 11). In this tree, branches marked in yellow are those containing PKs that possess ECTS. As indicated, PKs containing the ECTS are distributed across eight distinct groups throughout the phylogenetic tree. This sparse or patchy distribution is often interpreted as evidence for gene acquisition via lateral gene transfer (LGT) from prokaryotes. However, gene loss can generate the same patterns [33]. In this particular case, ECTS cannot be found as an isolated gene; instead, it is always found as the C-terminal domain of PKs. Therefore, it is not likely that ECTS could be due to LGT events. In contrast, loss of the ECTS domain seems to be a more probable event. Indeed, the fact that within a taxon that possesses PKs with an ECTS, we can also find PKs without ECTS suggests that this domain is prone to loss. This finding is in concordance with Bremer et al. [34], who found that the probability of losing a gene is surprisingly higher than that of local gene transfer. Nevertheless, evolutionary divergence cannot be ruled out due to the low identity of the ECTS between the phyletic groups that possess it. It is interesting to note that PKs possessing an ECTS can be found in both the K^+^-dependent and K^+^-independent branches. Thus, considering the overall data, it is likely that the ECTS is an ancestral feature found in the first PKs that was lost in the majority of them. Finally, it is worth noting that within the phylum Thermoplasmatota (which comprises *Tha*PK), we did not find PK sequences without ECTS. The mandatory presence of ECTS in this taxon might be related to the need to preserve this sequence to ensure the stabilization of the quaternary structure of the PKs belonging to the Thermoplasmatota phylum. In contrast, as mentioned before, the relevance of the presence of ECTS in *Gst*PK [26] and MRSA PK [11], from the Bacillota phylum, is unclear and remains unknown. If ECTS does not serve an essential function outside the Thermoplasmatota phylum, this could explain its tendency to be lost in other taxa.

Finally, an intriguing question is to determine how many residues of ECTS would be necessary to preserve its function in *Tha*PK. According to the global tree of the family of PK, which contains or does not contain the ECTS (Figure 11), this extra domain was found in several phyla of the bacteria and archaea domains. This ECTS did not exhibit a consensus sequence (see Figure 10A), but it is always formed by about 100 amino acid residues (see Table S1). The superimposed models of PKs from each of the three main phyla (Halobacteriota, Bacillota, and Thermoplasmatota) where this ECTS is present (see Figure 10B), all show a similar folding pattern (see Figure 6B). Taken together, these findings suggest that the presence of the complete ECTS is likely required for proper function. However, it would be of interest, as a subject for future research, to study partial constructs to explore what structural elements within the ECTS are required to preserve its function in *Tha*PK. In this context, it is worth noting that the salt bridges likely stabilizing the quaternary structure of *Tha*PK are formed between the εN group of K271 of the A domain of one chain and the carboxyl group of D462 in the ECTS of the contiguous chain. Since the ECTS spans residues 450 to 544, and D462 lies only 13 residues into the domain, it is possible that a partial construct containing the N-terminal portion of ECTS could still fold correctly and maintain functionality, without requiring the entire domain. However, any truncation may also affect the overall folding of the ECTS. Therefore, it is essential to structurally confirm that the various truncations constructed do not disrupt the folding of the full-length ECTS before drawing any conclusions about the protein domains or segments that are critical for its stabilizing function.

## 3. Materials and Methods

### 3.1. Chemicals

Imidazole, ammonium sulfate, HEPES, KCl, NH_4_Cl, NADH, MOPS, divalent cations, monovalent cations, allosteric modulators, the cyclohexylammonium salts of ADP and PEP, chloramphenicol, and LB medium were from Sigma-Aldrich Co. (St. Louis, MO, USA). Sodium Phosphate Monobasic, NaCl, and (CH_3_)_4_NCl were from T.J. Baker (Phillipsburg, NJ, USA). Ampicillin (GoldBio, St. Louis, MO, USA). SDS and BN-PAGE were from BIO-RAD (Hercules, CA, USA). NADH sodium salt was converted to the cyclohexylammonium salt by ion exchange following the protocol provided by the manufacturer (Sigma-Aldrich).

### 3.2. Cloning and Expression of WT-ThaPK

The pyruvate kinase gene (*Tha*PK, 1635 bp) from *Thermoplasma acidophilum* (GenBank accession NC_002578) was synthesized by GenScript (Piscataway, NJ, USA), incorporating *NdeI* and *BamHI* restriction sites at the 5′and 3′ ends, respectively. The gene was cloned into the pET15b vector, resulting in the construct *Tha*PK/pET15b.

The plasmid features a His_6_ tag at the N-terminus and a site for thrombin. The construct was transformed into competent BL21(DE3) Codon Plus-RIL cells. The plasmid was isolated and sequenced to verify the absence of mutations. The optimal expression of the gene was achieved by inducing with 0.6 mM isopropyl 1-thio-D-galactopyranoside and incubating for 15 h at 25 °C.

### 3.3. Cloning and Expression of ECTS Truncated-ThaPK

A truncated version of the *Tha*PK gene (1347 bp) was generated by PCR using the mutagenic oligonucleotide Rv: 5′-*ggatcc*ttaaacggccaccttgac-3′. In the sequence, the italicized region represents the new *BamHI* restriction site, while the bolded region indicates the newly introduced stop codon. This codon will interrupt the synthesis of the ECTS of the *Tha*PK gene that codifies for V451 to K544. The truncated *Tha*PK gene was amplified using an external T7 promoter primer and a mutagenic oligonucleotide, Rv. The PCR reaction was prepared with the following components: 100 ng of construct *Tha*PK/pET15b, 0.2 mM dNTPs (Thermo Scientific, Waltham, MA, USA), 100 ng of each oligonucleotide, 1.5 mM MgCl_2_, and 0.02 U/µL of Phusion High-Fidelity DNA Polymerase (Thermo Scientific, Cat. No F530S). The amplification was performed under the following cycling conditions: initial denaturation at 98 °C for 1 min, followed by 25 cycles of 98 °C for 30 s, 55 °C for 45 s, and 72 °C for 1 min, with a final extension at 72 °C for 5 min. The PCR product was cloned into the pJET 1.2/blunt vector (Thermo Scientific) and subsequently confirmed by automated DNA sequencing at the Unidad de Biología Molecular, Instituto de Fisiología Celular, UNAM (Mexico City, Mexico). The truncated *Tha*PK gene was subcloned into the pET15b vector after digestion with *Nde*I and *Bam*HI (New England BioLabs, Ipswich, MA, USA).

### 3.4. Cell Growth and Purification of WT-ThaPK and ECTS Truncated-ThaPK

LB medium containing 100 µg/mL ampicillin and 34 µg/mL chloramphenicol was inoculated either with *Tha*PKpET-15b or ECTS truncated*-ThaPK* into BL21(DE3) Codon Plus-RIL. Expression was induced at 25 °C with 0.6 mM isopropyl 1-thio-D-galactopyranoside at an OD of about 0.6. The enzymes were purified as in reference [2] with some modifications. After the cells were lysed by sonication, the suspension was centrifuged, and the supernatant was loaded onto a His Trap FF column. The enzyme was then eluted with a linear gradient of imidazole (10–500 mM). The fractions that exhibited PK activity were pooled and concentrated by membrane filtration (Centricon 100,000 MW) and desalted on a HiTrap Desalting column. The fractions with maximal PK activity were pooled and loaded on a HiTrap DEAE FF column, and the enzyme was eluted with a linear gradient of KCl (0–700 mM). To remove the His_6-_tag, a Thrombin Clean Cleave kit (SIGMA) was used. The thrombin resin was incubated with 1 mg of purified protein in a final volume of 1 mL, for up to 24 h. After incubation, aliquots of 0.1 mL were taken at different times, centrifuged, and the protein in the supernatant was expected to be His_6-_tag-free. After several attempts, the protein in the supernatants migrated in a SDS-PAGE (12%) identical to before the incubation with the thrombin resin. Therefore, thrombin could not cleave the tag, and this step was omitted. The enzyme was precipitated with ammonium sulfate at 80% saturation and stored at 4 °C. WT-*Tha*PK was approximately 90% pure, as determined by SDS-PAGE at 12% polyacrylamide, 4 °C, and 100V for 1.5 h [12]. To determine the oligomeric state, 500 µg of the enzyme was loaded onto a Superdex 200 (10/300 GL) GE Healthcare column (Uppsala, Sweden), which had been previously equilibrated with 50mM HEPES-NaOH, 150 mM NaCl, pH 7.5, and calibrated with the Gel Filtration standard from BIORAD (Hercules, CA, USA).

In the Section 2, Figure 9 shows a sample of *Tp*PK was used. It was grown, expressed, and purified as described in [2].

### 3.5. Assays of Pyruvate Kinase Activity

Ammonium sulfate-free enzymes of WT-*Tha*PK, truncated-*Tha*PK, and hog muscle lactate dehydrogenase (LDH) (ROCHE) were obtained as described in reference [35]. Contaminating NH_4_^+^, Na^+^, and K^+^ in reaction mixtures were below the detection limit (10 μM) as indicated previously in [36]. The formation of pyruvate was measured at 45 °C in a coupled system with LDH and NADH as described before [37]. The specific activity was not increased by the inclusion of 5-fold higher concentrations of LDH.

The reaction mixture contained 50 mM MOPS-(CH_3_)_4_NOH pH 6.5, the concentrations of phosphoenolypyruvate (PEP) and ADP, of divalent cations (Mg^2+^ and Mn^2+^), of monovalent cations (Li^+^, Na^+^, K^+^, NH4^+^, Rb^+^, and Cs^+^), of allosteric modulators (G6P, Fru 1,6-bis, R-5P, AMP, 3PG) and of the inhibitors (oxalate and AMP) are indicated in each figure legend. The MgADP complexes and free Mg^2+^ concentrations were calculated using the first original version of the software CHELATOR [38]. The MnADP complexes and Mn^2+^ concentrations were calculated using the K_d_ of Mn^2+^ [39]. The ionized PEP concentrations were calculated considering a pK value of 6.3 [40]. (CH_3_)_4_N^+^ was used to compensate for the varying substrate concentrations, thereby maintain a constant ionic strength of 200 mM. The concentrations of LDH were sufficient to overcome the inhibition by oxalate, and the inclusion of 5-fold higher concentrations did not increase the specific activities of PKs. The reaction mixtures were incubated for 10 min at 45 °C before initiating the reaction with a 2 min incubation of WT-*Tha*PK or the truncated-*Tha*PK enzyme at the same temperature.

### 3.6. Kinetic Studies

Initial velocities of WT-*Tha*PK were determined in the absence and presence of dead-end inhibitors (oxalate or AMP). In the former condition, the patterns were obtained at varying concentrations of PEP at several fixed levels of MgADP. In the latter condition, the inhibition patterns were obtained by varying one substrate with the second fixed at different levels of the inhibitor.

### 3.7. AlphaFold Model of WT-ThaPK

The structural model of the WT-*Tha*PK was obtained with the program Swissmodel online using as a template the model of AlphaFold “AF-P32044-F1 monomer” [41] (NCBI Reference Sequence: WP_010901306.1), which was obtained from the AlphaFold3 Server developed by DeepMind and EMBL-EBI (https://alphafoldserver.com/, accessed on 21 March 2025) [41]. The PDB file was downloaded from the following link: https://alphafoldserver.com/fold/183c1f1c2a9766cf (accessed on 21 March 2025).

### 3.8. Docking

Docking analysis was performed using the structural model of the WT-*Tha*PK, obtained from https://alphafold.ebi.ac.uk/entry/P32044, accessed on 21 March 2025 [41]. Structures of AMP and PEP were constructed and minimized using AVOGRADRO software version 1.97.0 (accessed on 01 August 2024) [42]. AutoDockTools 1.5.4 was used to prepare the PDB files of the protein and compounds. Polar hydrogen atoms and the Kollman united-atom partial charges were added to the protein structures. In contrast, Gasteiger-Marsili charges and rotatable groups were automatically assigned to the ligand structures. We use idock to run all the docking [43]. The grid box size was 60 Å × 62 Å × 62 Å in the x, y, and z dimensions, with central coordinates of −10.32 Å, 1.12 Å, and 11.26 Å for x, y, and z, respectively, and an exhaustiveness of 25. The best conformational states were visualized with PyMOL version 2.4.0 and Maestro Visualizer v.21.1.020298.

### 3.9. Circular Dichroism Experiments

CD spectra of WT-*Tha*PK and truncated-*Tha*PK were recorded in the far-UV range on a Jasco J715 spectropolarimeter. A 0.1 cm quartz cell was used. The protein solutions were prepared in 20 mM NaH_2_PO_4_-NaOH buffer, pH 7.0, with a protein concentration of 100 µg/mL. Spectral scans were run from 195 to 260 nm at 1 nm intervals, with three repetitions, and a time constant of 5 s. The experiments were conducted at 25 °C. The spectra of blanks were subtracted from those that contained the protein. CD is expressed as molar residue ellipticity.

### 3.10. Differential Scanning Calorimetry (DSC)

Differential scanning calorimetry experiments were carried out using a microcalorimeter instrument (VP-Capillary DSC, MicroCal, LLC, Northampton, MA, USA). The heat capacity measurements were performed at a heating rate of 1.5 °C/min in the temperature range 15 °C to 100 °C. The protein concentration was 1 mg/mL, and protein samples were prepared in a 50 mM MOPS-(CH_3_)_4_NOH solution at pH 6.5. To obtain the baseline, both the sample cell and the reference cell were filled with the 50 mM MOPS-(CH_3_)_4_NOH solution at pH 6.5. All experiments were conducted according to the procedures outlined in [2], with some modifications.

### 3.11. Molecular Dynamics Simulations

The structural model of the WT-*Tha*PK monomer was obtained from the AlphaFold3 Server (https://alphafoldserver.com/, accessed on 21 March 2025) [41]. For the Molecular dynamics simulations (MDS), the protein was first prepared with the pdb4amber script, part of AmberTools, and the GAFF force field was chosen for ligands [44]. Then, the coordinates and topologies of the complexes were prepared using the tLEAP module of AMBER23 [45,46,47]. Subsequently, hydrogen atoms and some missing atoms were added to the structure and the complex using the tLEAP module with the protein force field.ff19SB [47]; after that, an optimization of the hydrogen bond network was performed to increase the stability of the solute. At this point, Cl^−^ or Na^+^ counterions were included to neutralize the system. The complexes were solvated in an octahedral box of water molecules using the TIP3P model, with the box boundaries set 12 Å from the protein surface.

MDS were performed at 1 atm and 310.15 K, maintained with the Berendsen barostat and thermostat. Periodic boundary conditions and Ewald particle mesh sums (1 Å spacing) were used to treat long-range electrostatic interactions. To calculate direct interactions, a 10 Å cutoff was used. On the other hand, to satisfy the binding constraints, the SHAKE algorithm was employed, thereby allowing the use of a 2 fs time step to integrate Newton’s equations, as mentioned in the Amber 24 package. Amber force field parameters, protein.ff19SB, were also used for all residuals. The calculations were performed using a graphics processing unit-accelerated MD engine in AMBER (pmemd.cuda), a program package running entirely on CUDA-enabled GPUs [48,49]. Simulations were performed on an Ubuntu 22.04 Workstation with an NVIDIA Gigabyte GeForce RTX 4090 GPU, yielding a maximum performance of 316 ns/day.

This protocol begins with an initial structure minimization, followed by pressure equilibration at 315 K and 1.0 atm, respectively. Before MDS begins to produce, the system is equilibrated for 500 ps. Each complex produced 100 ns of MDS (in triplicate). The CPPTRAJ tool, as implemented in AMBER23 utilities, performed all analyses [32,46]. RMSD calculations were performed considering C, CA, and N. Graphs were created using Origin 2018. Chimera was used to visualize and create the MDS images [50,51].

### 3.12. 1D Blue Native-PAGE and 2D SDS-PAGE

One-dimensional BN-PAGE 1 mm mini gels were performed with a linear 4–18% acrylamide gradient according to [52]. The gels were run at 4 °C and 100 V until the protein sample had reached the separating gel. Afterwards, the gels were run at 9 mA. Two-dimensional SDS-PAGE 1.5 mm mini gels at 12% polyacrylamide were prepared according to [12]. The gels were run at 4 °C and 100 V. Protein concentrations were determined by measuring the absorbance at 280 nm using the absorptivity of 0.353, 0.564, and 0.335 mL mg^−1^ cm^−1^ for WT-*Tha*PK, *Tp*PK, and truncated-*Tha*PK, respectively, according to ProtParam (https://web.expasy.org/protparam accessed on 29 September 2022).

BN-PAGE gels were loaded with 50 µg of protein/well in the order indicated in Figure 9A. In lane 3, loaded with the truncated-*Tha*PK, most of the protein aggregated at the top of the gel, and three protein bands (indicated by black arrows) appeared as apparent smaller aggregation states of the enzyme. These smaller bands were resolved in 2D-SDS-PAGE, as shown in panel B3.11. Phylogenetic Analysis of a Sample of PK Sequences That Include the Domain of Extra C-Terminal Sequence (ECTS)

Non-redundant protein sequences from a previously published phylogenetic analysis [2] were used to identify PK amino acid sequences with an extra C-terminal sequence. Five clusters of PK sequences with an ECTS were identified. A sample of these sequences was used as bait to retrieve all available PK sequences with an ECTS using BlastP searches at the NCBI site [53] (https://www.ncbi.nlm.nih.gov/, accessed on 11 April 2025). Sequences selected as a bait were extra C-terminal sequences from PK (without A, B, and C domains) of: *Staphylococcus aureus* (NP_372221), *Geobacillus stearothermophilus* (Q02499), *Methanocella conradii* (H8I9P5), *Haloterrigena turkmenica* (D2RXQ5), *Nostoc* sp. (NP_488048), and *Ferroplasma acidarmanus* (S0APK2). Multiple amino acid sequence alignments were generated using the MUSCLE algorithm, as implemented in MEGA 12 [53,54], with a structural alignment constructed using the VAST algorithm as a guide [55], and then corrected manually using BioEdit [56]. We identified the evolutionary relationship in the ECTS domain (excluding A, B, and C domains) from partial and full-sequence PKs by constructing a rootless phylogenetic tree using the maximum likelihood method, with the Le-Gascuel model, as implemented in the MEGA 12 program [54]. Branching reliability was estimated using a 500-replicate bootstrap analysis.

## 4. Conclusions

In contrast to previous reports, we found that WT-*Tha*PK is a K^+^-independent enzyme, despite retaining three of the four conserved residues typically found in the K^+^-dependent PKs [1,2]. It is constitutively active, exhibiting neither cooperativity nor allosteric activation. WT-*Tha*PK displays a high affinity for its substrates, including one of the highest reported affinities for MgADP, and its catalytic efficiency is equally high in the presence of either Mg^2+^ or Mn^2+^. Like most PKs, it follows a rapid-equilibrium random-order mechanism. Structural models of both the monomer and tetramer were constructed, revealing that WT-*Tha*PK possesses an extended ECTS. The tetramer model showed three salt bridges formed between adjacent dimers: two involving interactions between the ECTS and the A domain, and one between the C domains. To investigate the role of this sequence, a truncated version of the enzyme lacking the ECTS was generated. Compared to the WT-*Tha*PK, the truncated enzyme exhibited less than 10% of the activity, significantly reduced affinity for PEP, indicative of a severely disrupted active site, and a notable loss of native interactions (approximately 80%), likely due to aggregation. Furthermore, it completely lost its tetrameric state. These results suggest that the ECTS plays a critical role in stabilizing the quaternary structure required for the enzyme to adopt its active conformation. It is worth noting that, despite the low sequence identity between the ECTS of *Tha*PK and those previously described in *Gst*PK, MRSA PK, or PKs from recently identified phyletic groups, the scattered distribution of PKs containing an ECTS suggests that the ancestral PK likely possessed this sequence. Throughout evolution, many PKs appear to have lost the ECTS. However, this loss has not been observed in PK sequences from the Thermoplasmatota phylum (e.g., *Tha*PK), where the ECTS is known to play an essential role. Notably, within Thermoplasmatota, we did not identify any PK sequences lacking the ECTS. The consistency of this sequence in this phylum may reflect the necessity to preserve it to ensure the stabilization of the quaternary structure of PKs in this lineage. In contrast, as previously mentioned, the functional relevance of the presence of the ECTS in *Gst*PK [26] and MRSA PK [11], both from the Bacillota phylum, remains unclear. If ECTS does not perform a critical function outside the Thermoplasmatota phylum, this could explain its frequent loss in other taxa.

## Figures and Tables

**Table 1:** Kinetic apparent parameters for PEP^3-^, MgADP, free Mg^2+^ of *Tha*PK. The data of Figure 2 were fitted (nonlinear regression Origin version 7.0) to the Michaelis-Menten equation *v* = V_max_ * [S]/*K_m_* + [S] or the Hill equation *v* = *V_max_* * [S]*^n^*/*K_0.5_^n^* + [S]*^n^*. The means and standard deviation from three experiments are shown. *K_app_* represents *K_m_* or *K_0.5_*.

Mg^2+^ Complexes					
Substrate	*K_app_* (mM)	*n*	*V_max app_* (µmol/min▪mg)	*k_cat_* (s^−1^)	Log *k_cat/_K* (s^−1^/M)
PEP^3^	0.066 ± 0.003	-	77 ± 1	315 ± 3	6.68
Mg-ADP	0.024 ± 0.001	-	75 ± 1	307 ± 5	7.10
Mg^2+^_free_	0.188 ± 0.018	1.8 ± 0.1	73 ± 1	299 ± 6	6.20

**Table 2:** Kinetic apparent parameters for PEP^3-^, Mn-ADP, free Mn^2+^ of *Tha*PK. The data of Figure 3 were fitted (nonlinear regression Origin version 7.0) to the Michaelis-Menten equation *v* = V_max_ * [S]/*K_m_* + [S] or the Hill equation *v* = *V_max_* * [S]*^n^*/*K_0.5_^n^* + [S]*^n^*. The means and standard deviation from three experiments are shown. *K_app_* represents *K_m_* or *K_0.5_*.

Mn^2+^ Complexes					
Substrate	*K_app_* (mM)	*n*	*V_max app_* (µmol/min▪mg)	*k_cat_* (s^−1^)	Log *k_cat/_K* (s^−1^/M)
PEP^3−^	0.018 ± 0.001	1.7 ± 0.1	33 ± 1	134 ± 1	6.87
Mn-ADP	0.011 ± 0.001	--	33 ± 1	137 ± 3	7.14
Mn^2+^_free_	0.082 ± 0.002	1.3 ± 0.1	25 ± 1	96 ± 1	6.08

**Table 3:** Intersecting patterns, kinetic mechanism, and kinetic constants of *Tha*PK. Intersecting patterns were taken from the double reciprocal plots of initial velocity data. The data shown in Figure 4 were globally fitted (nonlinear regression, Origin version 7.0) to the equation describing a rapid equilibrium random order mechanism *v* = *V_max_*[A][B]/(*K_a_K_b_* + *K_a_*[B] + *K_b_*[A] + [A][B], where *v* represents the initial velocity, A is PEP^3−^, B is MgADP and *K_a_* and *K_b_* are the Michaelis-Menten constants for PEP^3-^ and MgADP, respectively. Standard deviation values are shown. Catalytic efficiency values *k_cat_/K_m_* (s^−1^ M^−1^) are expressed in log form.

Substrate	Initial Velocity Intersecting Patterns 1/*v* Versus 1/[S]	Kinetic Mechanism	*V_max_* (µmol/min▪mg)	*K_m_* (mM)	*k_cat_* s^−1^	Log *k_cat_* /*K_m_* s^−1^M^−1^
PEP^3−^	Intersects to the left of the 1/*v* axis and on the 1/S axis	Random rapid equilibrium	106 ± 3	0.031 ± 0.002	418	7.14
ADP-Mg				0.023 ± 0.002		7.25

**Table 4:** Dead-end inhibition patterns and inhibition constants for oxalate and AMP in *Tha*PK. Inhibition patterns were taken from the double reciprocal plots of inhibition experiments (Figure 5). Inhibition constants were calculated from the fitness of the complete data set to the corresponding linear competitive inhibition equation (C) *v* = *V*_max_ * [S]/(*K_m_* (1 + [I]/*Ki*) + [S]) and linear mixed inhibition equation (MT) *v*= *V*_max_ * [S]/(*K_m_* (1 + [I]/*Ki*) + [S](1 + [I]/*αKi*), where α < 1 for MT; *Ki* is the inhibition constant.

Dead End Analog of PEP: Oxalate		Dead End Analog of Mg-ADP: AMP		*Ki* (Oxalate) µM	*Ki* (AMP) mM
1/*v* vs. 1/PEP, fixed Mg-ADP	1/*v* vs. 1/MgADP, fixed PEP	1/*v* vs. 1/PEP, fixed Mg-ADP	1/*v* vs. 1/Mg-ADP, fixed PEP		
C	MT	C	C	44 ± 1	3.3 ± 0.3

**Table 5:** Kinetic apparent parameters for PEP^3-^ and MgADP of the ECTS truncated-*Tha*PK. The saturation curves were fitted (nonlinear regression Origin version 7.0) to the Hill equation v = *V*_max_ * [S]*^n^/K_0.5_^n^* + [S]*^n^*, where S indicates the concentration of PEP^3−^ or MgADP. The means and standard deviation of three experiments are shown.

ECTS truncated-*Tha*PK					
Substrate	*K_0.5 app_* (mM)	*n*	*V_max app_* (µmol/min▪mg)	*k_cat_* (s^−1^)	Log *k_cat/_K* (s^−1^/M)
PEP^3−^	3.15 ± 1	1.7 ± 0.4	8.8 ± 1.9	29± 6	3.96
Mg-ADP	0.17 ± 0.01	2.1 ± 0.2	6.5 ± 0.2	21 ± 1	5.09

## Data Availability

The data that support the findings of this study are available from the corresponding author upon reasonable request.

## Supplementary Materials

The following supporting information can be downloaded at: https://www.mdpi.com/article/10.3390/ijms26178410/s1.

## References

[1] Oria-Hernández J., Riveros-Rosas H., Ramírez-Sílva L. (2006). Dichotomic phylogenetic tree of the pyruvate kinase family: K^+^-dependent and -independent enzymes. J. Biol. Chem..

[2] De la Vega-Ruíz G., Domínguez-Ramírez L., Riveros-Rosas H., Guerrero-Mendiola C., Torres-Larios A., Hernández-Alcántara G., García-Trejo J.J., Ramírez-Silva L. (2015). New insights on the mechanism of the K^+^-independent activity of crenarchaeota pyruvate kinases. PLoS ONE.

[3] Göker M., Oren A. (2024). Valid publication of names of two domains and seven kingdoms of prokaryotes. Int. J. Syst. Evol. Microbiol..

[4] Potter S., Fothergill-Gilmore L.A. (1992). Purification and properties of pyruvate kinase from *Thermoplasma acidophilum*. FEMS Microbiol. Lett..

[5] Johnsen U., Reinhardt A., Landan G., Tria F.D.K., Turner J.M., Davies C., Schónheit P. (2019). New views on an old enzyme: Allosteric regulation and evolution of archaeal pyruvate kinases. FEBS J..

[6] Ramírez-Silva L., Hernández-Alcántara G., Guerrero-Mendiola C., González-Andrade M., Rodríguez-Romero A., Rodríguez-Hernández A., Lugo-Munguía A., Gómez-Coronado P.A., Rodríguez-Méndez C., Vega-Segura A. (2022). The K^+^-Dependent and -Independent Pyruvate Kinases Acquire the Active Conformation by Different Mechanisms. Int. J. Mol. Sci..

[7] Sakai H., Ohta T. (1993). Molecular cloning and nucleotide sequence of the gene for pyruvate kinase of Bacillus stearothermophilus and the production of the enzyme in Escherichia coli. Evidence that the genes for phosphofructokinase and pyruvate kinase constitute an operon. Eur. J. Biochem..

[8] Tanaka K., Sakai H., Ohta T., Matsuzawa H. (1995). Molecular cloning of the genes for pyruvate kinase of two bacilli, Bacillus psychrophilus and Bacillus licheniformis, and comparison of the properties of the enzymes produced in Escherichia coli. Biosci. Biotechnol. Biochem..

[9] Muñoz M.E., Le Borgne S., Bolívar F., Valle F. (1997). Molecular cloning of the gene that codes for the pyruvate kinase of Bacillus subtilis, primary characterization of a strain carrying this gene insertionally inactivated. Rev. Latinoam. Microbiol..

[10] Muñoz M.E., Ponce E. (2003). Pyruvate kinase: Current status of regulatory and functional properties. Comp. Biochem. Physiol. B.

[11] Zoraghi T., Swayze R., Finlay B.B., Brunham R.C., McMaster W.R., Reiner N.E. (2010). Functional analysis, overexpression and kinetic characterization of pyruvate kinase from Methicillin-Resistant *Staphylococcus aureus*. Biochemistry.

[12] Laemmli U.K. (1970). Cleavage of structural proteins during the assembly of the head of bacteriophage T4. Nature.

[13] Guerrero-Mendiola C., García-Trejo J.J., Encalada R., Saavedra E., Ramírez-Silva L. (2017). The contribution of two isozymes to the pyruvate kinase activity of Vibrio cholerae: One K^+^-dependent constitutively active and another K^+^-independent with essential allosteric activation. PLoS ONE.

[14] Ramírez-Silva L., Guerrero-Mendiola C., Cabrera N. (2014). The Importance of Polarity in the Evolution of the K^+^ Binding Site of Pyruvate Kinase. Int. J. Mol. Sci..

[15] Kayne F.J. (1971). Thallium (I) activation of pyruvate kinase. Arch. Biochem. Biophys..

[16] Ramírez-Silva L., Oria J., Gómez-Puyou A., Tuena de Gómez-Puyou M. (1997). The contribution of water to the selectivity of pyruvate kinase for Na^+^ and K^+^. Eur. J. Biochem..

[17] Mildvan A.S., Cohn M. (1965). Kinetic and magnetic resonance studies of the pyruvate kinase reaction: I. Divalent Metal Complexes of Pyruvate Kinase. J. Biol. Chem..

[18] Dupont C.L., Butcher A., Vaalas R.E., Bourne P.E., Caetano-Anallés G. (2010). History of biological metal utilization inferred through phylogenomic analysis of protein structures. Proc. Natl. Acad. Sci. USA.

[19] Reynard A.M., Hass L.F., Jacobsen D.D., Boyer P.D. (1961). The correlation of reaction kinetics and substrate binding with the mechanism of pyruvate kinase. J. Biol. Chem..

[20] Ainsworth S., MacFarlane N. (1973). A kinetic study of rabbit muscle pyruvate kinase. Biochem. J..

[21] Oria-Hernández J., Cabrera N., Pérez-Montfort R., Ramírez-Silva L. (2005). Pyruvate kinase revisited. The activating effect of K^+^. J. Biol. Chem..

[22] Cleland W.W. (1970). The Enzymes.

[23] Reed G.H., Morgan S.D. (1974). Kinetic and magnetic resonance studies of the interaction of oxalate with pyruvate kinase. Biochemistry.

[24] Waterhouse A., Bertoni M., Bienert S., Studer G., Tauriello G., Gumienny R., Heer F.T., de Beer T.A.P., Rempfer C., Bordoli L. (2018). SWISS-MODEL: Homology modelling of protein structures and complexes. Nucleic Acids Res..

[25] Pocalyko D.J., Carroll L.J., Martin B.M., Babbitt P.C., Dunaway-Mariano D. (1990). Analysis of sequence homologies in plant and bacterial pyruvate phosphate dikinase, enzyme I of the bacterial phosphoenolpyruvate: Sugar phosphotransferase system and other PEP-utilizing enzymes. Identification of potential catalytic and regulatory motifs. Biochemistry.

[26] Sakai H. (2004). Possible structure and function of the extra C-terminal sequence of pyruvate kinase from Bacillus stearothermophilus. J. Biochem..

[27] Suzuki K., Ito S., Shimizu-Ibuka A., Sakai H. (2008). Crystal Structure of Pyruvate Kinase from *Geobacillus stearothermophilus*. J. Biochem..

[28] Zoraghi R., Worrall L., See R.H., Strangman W., Popplewell W.L., Gong H., Samaai T., Swayze R.D., Kaur S., Vuckovic M. (2011). Methicillin-resistant *Staphylococcus aureus* (MRSA) Pyruvate Kinase as a Target for Bis-indole Alkaloids with Antibacterial Activities. J. Biol. Chem..

[29] Adasme M.F., Linnemann K.L., Bolz S.N., Kaiser F., Salentin S., Haupt V.J., Schroeder M. (2021). PLIP 2021: Expanding the scope of the protein–ligand interaction profiler to DNA and RNA. Nucleic Acids Res..

[30] Geenfield N. (2006). Using circular dichroism spectra to estimate protein secondary structure. Nat. Protoc..

[31] Goyal M., Chaudhuri T.K., Kuwajima K. (2014). Irreversible denaturation of methanodextrin glucosidase studied by differential scanning calorimetry, circular dichroism, and turbidity measurements. PLoS ONE.

[32] Roe D.R., Cheatham T.E. (2013). PTRAJ and CPPTRAJ: Software for Processing and Analysis of Molecular Dynamics Trajectory Data. J. Chem. Theory Comput..

[33] Bremer N., Martin W.F., Steel M. (2025). Surprising effects of different loss in genome evolution: The last-one-out. FEMS Microbiol. Lett..

[34] Altschul S.F., Madden T.M., Schäffer A.A., Zhang J., Zhang Z., Miller W., Lipman D.J. (1997). Gapped BLAST and PSI-BLAST: A new generation of protein database search programs. Nucleic Acids Res..

[35] Kasahara M., Penefsky H.S. (1978). High affinity binding of monovalent Pi by beef heart mitochondrial adenosine triphosphatase. J. Biol. Chem..

[36] Ramírez-Silva L., de Gómez-Puyou M.T., Gómez-Puyou A. (1993). Water-induced transitions in the K^+^ requirements for the activity of pyruvate kinase entrapped in reverse micelles. Biochemistry.

[37] Büchner T., Pleiderer G. (1955). Methods in Enzymol.

[38] Schoemakers J.M., Visser G.J., Flik G., Theuvenet P.R. (1992). CHELATOR: An improved method for computing metal ion concentrations in physiological solutions. Biotechniques.

[39] Susan-Resiga D., Nowak T. (2004). Proton donor in yeast pyruvate kinase: Chemical and kinetic properties of the active site Thr298 to Cys mutant. Biochemistry.

[40] Dougherty T.M., Cleland W.W. (1985). pH Studies of the chemical mechanism of rabbit muscle pyruvate kinase. 2. Physiological substrates and phosphoenol-αketobutyrate. Biochemistry.

[41] Abramson J., Adler J., Dunger J., Evans R., Green T., Pritzel A., Ronneberger O., Willmore L., Ballard A.J., Bambrick J. (2024). Accurate structure prediction of biomolecular interactions with AlphaFold 3. Nature.

[42] Hanwell M.D., Curtis D.E., Lonie D.C., Vandermeersch T., Zurek E., Hutchison G.R. (2012). Avogadro: An advanced semantic chemical editor, visualization, and analysis platform. J. Cheminform..

[43] Masters L., Eagon S., Heying M. (2020). Evaluation of consensus scoring methods for AutoDock Vina, smina and idock. J. Mol. Graph. Model..

[44] Wang J., Wolf R.M., Caldwell J.W., Kollman P.A., Case D.A. (2004). Development and testing of a general amber force field. J. Comput. Chem..

[45] Case D.A., Aktulga H.M., Belfon K., Cerutti D.S., Cisneros G.A., Cruzeiro V.W.D., Forouzesh N., Giese T.J., Gotz A.W., Gohlke H. (2023). AmberTools. J. Chem. Inf. Model..

[46] Case D.A., Cheatham T.E., Darden T., Gohlke H., Luo R., Merz K.M., Onufriev A., Simmerling C., Wang B., Woods R.J. (2005). The Amber biomolecular simulation programs. J. Comput. Chem..

[47] Walker R.C., Crowley M.F., Case D.A. (2008). The implementation of a fast and accurate QM/MM potential method in Amber. J. Comput. Chem..

[48] Krieger E., Vriend G. (2015). New ways to boost molecular dynamics simulations. J. Comput. Chem..

[49] Salomon-Ferrer R., Gotz A.W., Poole D., Le Grand S., Walker R.C. (2013). Routine Microsecond Molecular Dynamics Simulations with AMBER on GPUs. 2. Explicit Solvent Particle Mesh Ewald. J. Chem. Theory Comput..

[50] Maruyama Y., Igarashi R., Ushiku Y., Mitsutake A. (2023). Analysis of Protein Folding Simulation with Moving Root Mean Square Deviation. J. Chem. Inf. Model..

[51] Pettersen E.F., Goddard T.D., Huang C.C., Couch G.S., Greenblatt D.M., Meng E.C., Ferrin T.E. (2004). UCSF Chimera- a visualization system for exploratory research and analysis. J. Comput. Chem..

[52] Schägger H., von Jagow G. (1991). Blue native electrophoresis for isolation of membrane protein complexes in enzymatically active form. Anal. Biochem..

[53] Edgar R.C. (2022). Muscle5: High-accuracy alignment ensembles enable unbiased assessments of sequence homology and phylogeny. Nat. Commun..

[54] Kumar S., Stecher G., Suleski M., Sanderford M., Sharma S., Tamura K. (2024). Molecular Evolutionary Genetics Analysis Version 12 for adaptive and green computing. Mol. Biol. Evol..

[55] Madej T., Marchler-Bauer A., Lanczycki C., Zhang D., Bryant S.H. (2020). Biological Assembly Comparison With VAST. Methods Mol. Biol..

[56] Hall T.A. (1999). BioEdit: A user-friendly biological sequence alignment editor and analysis program for Windows 95/98/NT. Nucleic Acids Symp. Ser..

