# Supplementary material for: The Functional State of Thermoplasma acidophilum Pyruvate Kinase Relies on an Extra Carboxyl-Terminal Sequence
Source: Int J Mol Sci. 2025 Aug 29;26(17):8410. doi: 10.3390/ijms26178410 (PMC12428139; doi:10.3390/ijms26178410)
Supplement: Supplementary file 1 [file ijms-26-08410-s001.zip › ijms-3770067-supplementary.pdf]

Supplementary Materials.

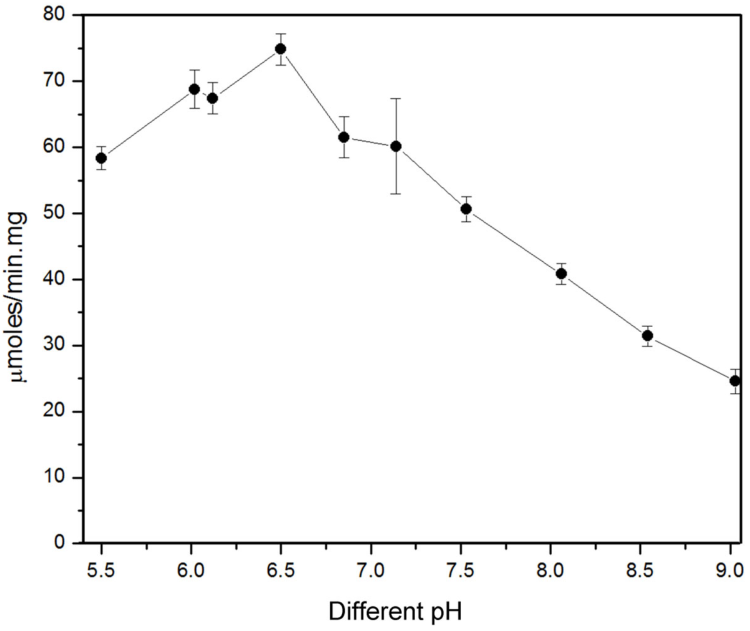

Figure S1. pH profile of *Tha*PK. The figure shows the activities of *Tha*PK determined in V_{max} conditions at each pH. For pHs 5.5, 6, 6.12, 6.5, 6.85, 7.14, and 7.53, the activities were determined in the presence of 3 mM PEP; whereas for pHs 8.06, 8.54, and 9, the activities were determined with 5 mM PEP. Besides the saturating PEP concentration, the reaction mixture contained 1 mM ADP, 7.2 mM Mg^{2+}, 2 mM AMP, 0.2 mM NADH, 10 µg/ml LDH, and 90 mM $(CH_3)_4N^+$ instead of 7.2 mM K^+ as proposed by Potter and Fothergill (1992) [4]. 50 mM MES was used in pHs 5.5 to 6.5, 50 mM HEPES was used in pHs 6.85 to 8.06, and 50 mM TRIS-HCl was used in pHs 8.54 and 9.03. The mean and standard deviations from three different experiments are shown. The experiments were carried out at 45ºC.

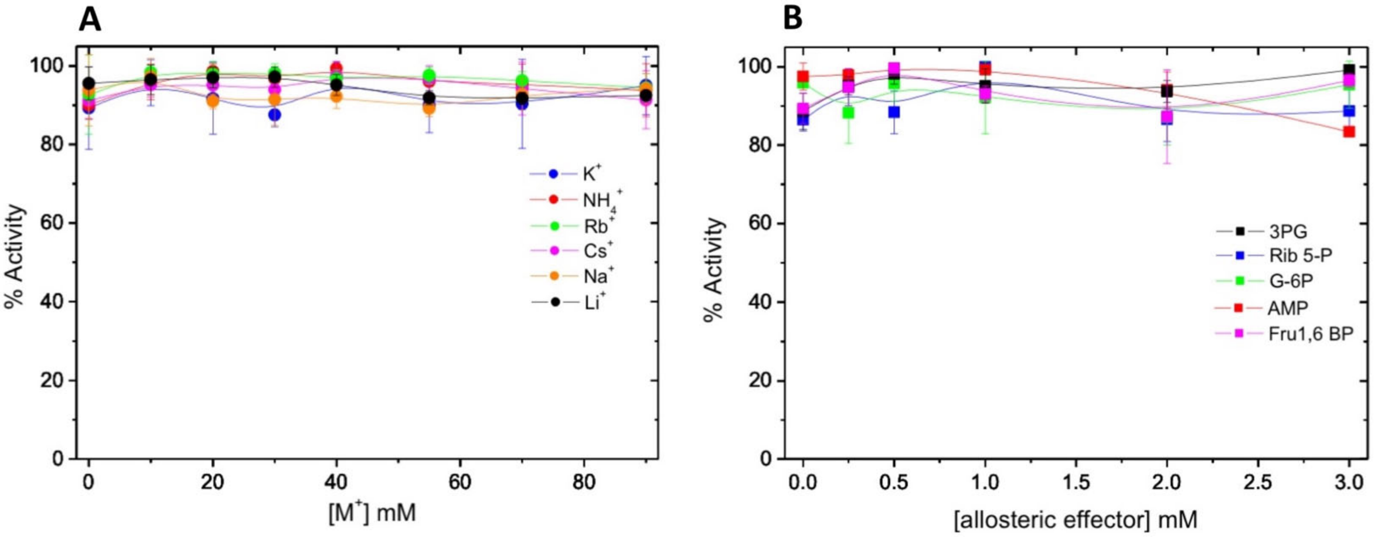

Figure S2. Effect of monovalent cations (A) and allosteric effectors (B) on the activity of WT-*Tha*PK. The activities of WT-*Tha*PK were determined in the presence of the indicated concentration of monovalent cation (A) or allosteric effector (B). The reaction mixture contained 50 mM MOPS pH 6.5, ADP 0.57 mM, 1.13 mM PEP in (A) and 0.066 mM ($K_{m\text{PEP}}$) PEP in (B), 1.66 mM Mg^{2+}_{total}, 1.41 mM Mg^{2+}_{free}, 0.2 mM NADH, 10 µg/ml LDH and ionic strength was maintained constant at 200 mM with $(CH_3)_4N^+$. Activities of *Tha*PK in the absence of M^+ or effector were ~75 and ~35 µmoles/min·mg, respectively. The mean and standard deviations from three different experiments are shown. The experiments were carried out at 45ºC.

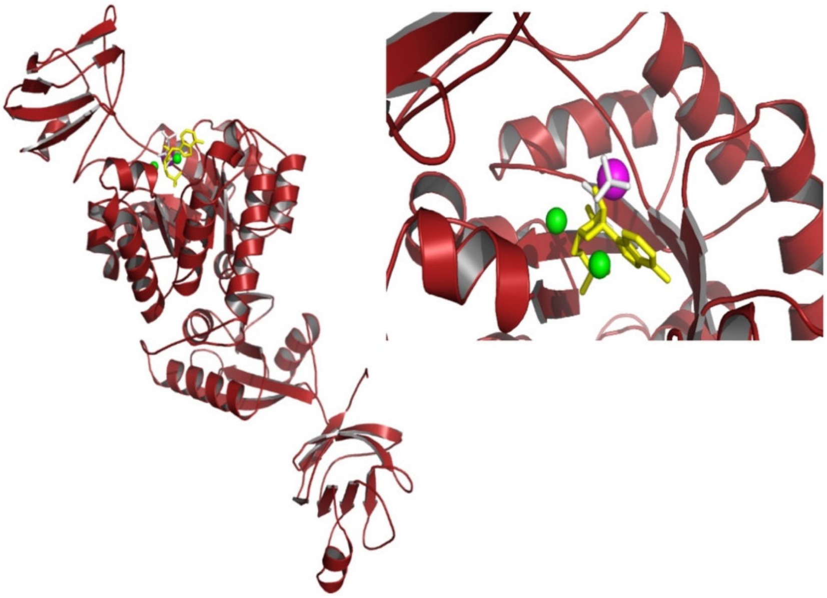

Figure S3. Molecular docking of WT-*Tha*PK with PEP, AMP, $Mg^{2+}$and K^{+}. The green and magenta spheres represent Mg^{2+} and K^{+}, respectively. The skeletons of AMP and PEP are shown in yellow and white sticks, respectively.

Table S1. Alignment of pyruvate kinase sequences that contained an extra carboxyl-terminal sequences (ECTS). Only the ECTS are displayed.

		10 20 30 40 50 60	
E_M0GLR0_HALL2	585	EPVATGRKIVGGRVAG-------PLYRTEDGDLTDVPEGAVLALSSEFDGEFDGDADKLA	53
E_D4GUB6_HALVD	585	EPVATGRKIVGGRVAG-------PLYRTEDGDLTDVPEGAVLALSSEFDGEFDGDADKLA	53
E_M0I6D0_9EURY	585	EPVATGRKIVGGRVAG-------PLYRTEDGDLTDVPEGAVLALSSEFDGEFDGDADKLA	53
E_M0FFY9_9EURY	585	EPVATGRKIVGGRVAG-------PLYRTEDGDLTDIPEGAVLALSSEFDGEFNGDADKLA	53
E_M0FY16_9EURY	585	EPVATGRKIVGGRVAG-------PLYRTEDGDLTDIPEGAVLALSSEFDGEFNGDADKLA	53
E_M0FS45_9EURY	585	EPVATGRKIVGGRVAG-------PLYRTEDGDLTDIPEGAVLALSSEFDGEFNGDADKLA	53
E_M0IYL0_9EURY	585	EPVATGRKIVSGRVAG-------PLYRTEDGDLTDVPEGAILALSAEFDGEFDGDADKLA	53
E_M0IA90_9EURY	585	EPVATGRKIVSGRVAG-------PLYRTEDGDLTDVPEGAILALSAEFDGEFDGDADKLA	53
E_M0HJT1_9EURY	586	EPVATGRKIVSGRVAG-------PLYRTEDGDLTDVPEGAILALSAEFDGEFDGDADKLA	53
E_M0GLR8_9EURY	586	EPVATGRKIVSGRVAG-------PLYRTEDGDLTDVPEGAILALSAEFDGEFNGDADKLA	53
E_M0I4Q1_9EURY	585	EPIATGRKIVSGRVAG-------PLYRTEDGDLSDVPEGAVLALSAEFDGEFDGDADKLA	53
E_M0IZU3_HALMT	585	EPIATGRKIVSGRVAG-------PLYRTEDGDLSDVPEGAVLALSAEFDGEFDGDADKLA	53
E_I3R2N6_HALMT	607	EPIATGRKIVSGRVAG-------PLYRTEDGDLSDVPEGAVLALSAEFDGEFDGDADKLA	53
E_M0GPJ1_9EURY	586	ESVANGRKVVGGRVAG-------PVYRTSDGDLSDVPEGAILSLTADFDGEFDGDADKLA	53
E_M0HUA5_9EURY	586	ESVANGRKVVGGRVAG-------PVYRTSDGDLSDVPEGAILSLAADFDGEFDGDADKLA	53
E_E4NRC8_HALBP	586	EAVATGRKVVGGRISG-------PLAITSDGDLSDVPDGALLALPAEFDGEFHGDAKKLG	53
E_M0CVL5_9EURY	594	EAVATGRKVVGGRVAG-------PLAVSDDGDLSDVPEGALLALTAAFDGEFDGDAKKLG	53
E_Q18JV3_HALWD	586	ETIATGRKVVGGRTAG-------PLATPTDGRLDEIPHGAILALPADFDAEFDHDAAMLG	53
E_G0LGR5_HALWC	586	ETIATGRKVVGGRTAG-------PLATPTDGRLDEIPHGAILALPADFDAEFDHDAAMLG	53
E_J3EZ11_9EURY	584	EAVATGRKVVGGRISG-------PLYRVTDGELRGVPDGAILAVPASFEGEFTGDVKKIS	53
E_E7QTI2_9EURY	582	ETIATGRSVVRGQTVG-------PAVRSSTGDLSEVPSGGILVLEPDFDGEFTGDTSRLA	53
E_R4W8G0_9EURY	594	ETVATGRSVVDGRVSG-------PVATAPDGDLGAVAEGSVLVLDSEFEAEFSGDPARLA	53
E_M0N190_9EURY	584	DIVASGRSIVEGVATG-------PLHHAEHGDLTSVPDGSVLMLPTDFDDEFTGDPSSLA	53
E_M0M4X2_HALMO	584	DIVASGRSIVDGVATG-------PLHHAEHGDLTSVPDGSVLTLPTDFDDEFTGDPSSLA	53
E_M0M9D2_9EURY	589	EIVASGRPVVSGHATG-------PLYHSTDGDLTDLPAGAILALPVDFDDEFVGDPSTLA	53
E_M0M5X3_9EURY	581	EVLASGRSVVTGRVAG-------PLHHAPDGDLTTVPEGAVLFVSQTFDGEFAGDPRKLG	53
E_M0MKB2_9EURY	580	EILATGRSVVDGRVSG-------PLSRTVDGDLTDLPEGTILSLPADFDDEFDGNSARLG	53
E_M0N0N7_9EURY	580	EILATGRSVVDGRVSG-------PLSRTVDGDLTGLSEGTILSLSADFDDEFDGDPTRLG	53
E_F7PNS4_9EURY	582	ESVATGQSVVDGLVTG-------PLVRTTDGDLESVPEGAILAVPEDFDGEFLGDPETLG	53
E_C7NP64_HALUD	582	ETVTTGQSVVDGLVTG-------PLVRTTDGDLESVPEGAILSVPADFDDEFLGDPSKLG	53
E_C7NYS9_HALMD	581	ERIANGRSVVDGLVSG-------PVYHVPDGDLSDVPDGAVISIAADFDGEFDGEVERIG	53
E_M0CQS7_9EURY	590	ETIASGRSVVEGLVSG-------ELHWTEDGDLSAVPEGAILAVPEDFDGEFTGDIEKLG	53
E_G0HY69_HALHT	596	ETVASGRSIVDGLVTGPVHRISTAPPRESPGDLSNVPDGAILAVPEGFDGEFVGDTSRIG	60
E_M0JGH6_HALVA	596	ETVASGRSIVDGLVTGPVHRISTAPPRESPGDLSNIPDGAILAVPEGFDGEFVGDTSRIG	60
E_M0LC35_HALJP	596	ETVASGRSIVDGLVTGPVHRISTAPPRESPGDLSNVPDGAILAVPEGFDGEFVGDTSRIG	60

```
E_M0KPP3_9EURY|596            ETVASGRSIVDGLVTGPVHRISTAPPQESPGDLSNVPDGAILAVPEGFDGEFIGDTSRIG 60
E_M0KV41_HALAR|596            ETVASGRSIVDGLVTGPVHRISTAPPRESPGDLSNVPDGAILAVPEGFDGEFVGDTSRIG 60
E_Q5V4I8_HALMA|610            ETVASGRSIVDGLVTGPVHRISTAPPRESPGDLSNVPEGAILAVPEGFDGEFVGDTSRIG 60
E_M0JZ82_9EURY|596            ETVASGRSIVDGLVTGPVHRISTAPPRESPGDLSNVPEGAILAVPEGFDGEFVGDTSRIG 60
E_M0K0G8_9EURY|610            ETVASGRSIVDGLVTGPVHRISTAPPRESPGDLSNVPEGAILAVPEGFDGEFVGDTSRIG 60
E_M0BVS9_9EURY|583            DTIETGRVVVEGRASG-------PIAHAPEGDLTDVPDGAIVAIDG--IDEFTGDVSKLA 51
E_L0I815_HALRX|583            DTIETGRVVVEGRASG-------PIAHAPEGDLTDVPDGAIVAIDA--IDEFTGDVSKLA 51
E_D8J7J0_HALJB|594            ETLETGTGVVSGRTSG-------PVVHTEDGDLAGVEPGAILSLPAGFDGEFTGELSRVG 53
E_Q9HSA5_HALSA|579            ETLSTGRAVVDGRTTG-------RTYRADSGDLSDAPENAVVLLAHGFDGEFDGDLSKIG 53
E_B0R347_HALS3|579            ETLSTGRAVVDGRTTG-------RTYRADSGDLSDAPENAVVLLAHGFDGEFDGDLSKIG 53
E_G4IHY5_9EURY|577            ETLASGRSVVDGRATG-------TATHVEDGDLADVPEGAVLVLDSTFDDEFSGDLSKVA 53
E_M0B9C3_9EURY|584            EALTTGRVVVEGRATG-------PLARVQDGDLTDVPEGAILALPTEFDAEFTGDVSKIG 53
E_L9ZS75_9EURY|584            EALTTGRVVVEGRATG-------PLARVQDGDLTDVPEGAILALPTEFDAEFTGDVSKIG 53
E_M0AKF4_NATA1|584            EALTTGRVVVEGRATG-------PLARVQDGDLTDVPEGAILALPTEFDAEFTGDVSKIG 53
E_D3SYX2_NATMM|588            EALTTGRVVVEGRATG-------PLARVPDGDLTDVPDGAIIALPADFDEEFTGDLNTIG 53
E_M0AAI6_9EURY|593            EALTTGRVVVGGRATG-------PVARVPDGDLTDVPEGAIIVLPSGFDEEFTGDLHTIG 53
E_L9ZVH2_9EURY|593            EALTTGRVVVGGRATG-------PVARVPDGDLTDVPDGAIIALPSEFDEEFTGDLHTIG 53
E_D2RXQ5_HALTV|585            EALTTGRVVVDGRVTG-------PLVELTDGDLTDVPEGAILSLPTDFDEEFTGDPTKIG 53
E_M0BWI7_9EURY|585            EALTTGRVVVDGRVTG-------PVVELTDGDLTDVPEGAILSLPTDFDEEFTGDPTKIG 53
E_L9WF02_9EURY|585            EALTTGRVVVEGRATG-------PLVHLSDGDITDIPDGAILSLPADFDEQFEGDPTRIG 53
E_L9W4T0_9EURY|585            EALTTGRVVVEGRATG-------PLVRLTDGDITEIPDGAILSLPADFDEQFEGDPTRIG 53
E_L9W3Q0_9EURY|599            EALTTGRVVVEGRTTG-------PLVRMTDGDLSDVPEGAVLSLAADFDEEFEGDLTTIG 53
E_L9XBF4_9EURY|585            EALTTGRVVVEGRVTG-------PVAELSDGDLSRVPEGAILSLSADYDEEFEGDLTKIG 53
E_F8D381_HALXS|585            EALTTGRVVVEGRATG-------PLVHLTDGDLSDVPDGAIVAVPSDFDEEFEGDLEKIA 53
E_L9XI31_9EURY|586            DALTTGRVVVEGRTTG-------PVVRLRDGDLSSIPDGAIVALGTAFDDEFEGDLERIG 53
E_L0JWJ5_9EURY|586            DALTTGRVVVEGRTTG-------PIARLHSGDLSTIPDGAIVALESEFDDEFEGDLDRLG 53
E_M0MBJ8_9EURY|593            EALTTGRVVVDGRATG-------PVARAPDGDLSDVPEGAILALPADFDGEFDGDVSKIG 53
E_M0L2S3_9EURY|605            EALTTGRVVVDGRATG-------PIARTTDGDLSEVPEGAILALPADFDEEFDGDVSKIG 53
E_L0ADH0_NATGS|585            EALTTGRVVVDGCATG-------PVTRVPGGDLSDVPDGAILALPAEFDDEFDGDPTKIG 53
E_M0BLU5_9EURY|585            DALTTGQVVVEGRATG-------PIVHATDGDLSGVPEGAILALSADFDAEFSSETDRIG 53
E_L0JRU4_NATP1|585            DALTTGQVVVEGRATG-------PIVHATDGDLSGVPEGAILALSADFDAEFSSETDRIG 53
E_M0CUB6_9EURY|585            DTLTTGQVVVEGRATG-------PVVHLSDGDLSAVPDGAIVALPADFDEEFSGETSRLG 53
E_L9YBC2_9EURY|585            DALTTGQVVVEGRATG-------PVVHVSDGDLSDIPEGAILALPADFDEEFTGDTSRIG 53
E_L9YIB5_9EURY|585            DALTTGQVVVEGRSTG-------PVVRVTDGDLSGVPDGAILALPADFDAEFTGDTSRLG 53
E_L9ZZY6_9EURY|585            DALTTGQVVVEGRSTG-------PVVRVTDGDLSGVPDGAILALPADFDAEFAGDTSRLG 53
E_L9YUZ4_9EURY|585            DTLTTGQVVVEGRSTG-------PVVRVTDGDLSGVPDGAILALPADFDAEFTGDTSRLG 53
E_I7C1X6_NATSJ|585            DALTTGQVVVEGRSTG-------PVVRVTDGDLSGVPDGAILALPGDFDAEFTGDTSRLG 53
E_G2MMV7_9ARCH|599            AVLASGRAAAEGYASG-------PVYRVANGDLTGLPDGAIAYLPGELEGGFTGDPTTLA 53
E_M0PIF3_9EURY|585            ETLVTGKAVVAGRIAA-------PVYRSADGDLSDLPEGSIVALGPGFEGEFSGDLDAVA 53
E_M0NXB3_9EURY|585            ETLVTGKAAVTGRIAA-------PVHRTDDGDLSGIPEGSIVALGPGFEGEFSGDLDAMA 53
E_B9LTN0_HALLT|585            ETLVTGKAVVAGRVAA-------PVYRTADGDLSDVPEGSVVALGPSFEGEFTGDLDAIA 53
E_M0DSX7_9EURY|585            ETLVTGKAVVAGSVAA-------PVYRTADGDLREVPEGSIVALGPNFEGEFTGDLDAVA 53
E_M0NZ95_9EURY|585            ETLVTGKAVVAGRVAA-------PVYRTADGDLSDVPDGSIVALGPNFEGEFTGDLDALA 53
E_M0DB95_9EURY|585            ETLVTGKAVVAGRVAA-------PVHRTEDGDIDALPDGSVVAIGSDFDGEFSGDLDRIA 53
E_M0FJ51_9EURY|585            ETLVTGKAVVAGRVAA-------PVHRTEDGDIDALPDGSVVAIGSDFDGEFSGDLDRIA 53
E_M0EM72_9EURY|585            ETLVTGKAAVAGRVAA-------PVHRTEDGDLDALPDGSVVALDADFDGEFSGDLDRIA 53
E_M0EC36_9EURY|585            ETVVTGKGAVAGRVAA-------PVHRTADGDLDALPEDSVVALGPDFDGEFAGDLDRIA 53
E_M0DGP4_9EURY|585            ETLVTGKAVVAGRVAG-------PVHRTADGDLDALPDGAVVALGADFDGEFSGDLDRIA 53
E_M0PND9_9EURY|585            ETLVTGKAVVAGRVAG-------PVHRTADGDLDALPDGAVVALGADFDGEFSGDLDRIA 53
E_M0NY16_9EURY|585            ETLVTGKAVVAGRVAG-------PVHRTADGDLDALPDGAVVALGADFDGEFSGDLDRIA 53
E_M0F352_9EURY|585            ETLVTGKAVVAGRVAG-------PVHRTADGDLDALPDGAVVALGADFDGEFSGDLDRIA 53
E_M0ER95_9EURY|585            ETLVTGKAVVAGRVAG-------PVHRTADGDLDALPDGAVVALGADFDGEFSGDLDRIA 53
E_Q3ISC3_NATPD|593            ERLATGRGVVSGRSAG-------PLVRTDDGDLSGVQSGAIVALPAGFDGEFDGDVTNIG 53
E_A7U0W4_9EURY|577            EVLASGRGVRTGRAAG-------PVVHSADGDLTGIPDGAVLVLPEAFDGELGGDLSAVG 53
K_Q5NW05_9ARCH|583            KELARGIGVGKDIVKG-------VARVIKSPSEFTNIRSGDILVIKEVDVDHIDDVKPAR 53
E_M1XZK5_9EURY|580            EVLASGRGVCAGRAVG-------PALVAPDGDLAGCPDGAVVVLPGDFEGEFTGDLAAIG 53
K_Q0W8N0_UNCMA|583            KELARGIGVGKDIVKG-------VARVIKSPSEFTNIRSGDILVIKEVDVDHIDDVKPAR 53
K_H8I9P5_METCZ|583            KEMAQGMGIGKGIVKG-------LVKVITTPADFGQIEKGDIIAIKEANIDRIDDIKKAS 53
K_D1YYS4_METPS|583            KEMAKGMGIGKGVVKG-------IIRIIASPEDFGKIQKGDIIAIKEADIDRIGDIKKAS 53
E_NP_623403_THETEN|583        DVILRGIGIGSKSTTG------TVCIIHDIGKDRDKFREGDIIVTTKTERDFMPLIEKAS 54
E_NP_783015_CLOTET|584        DILVQGRGAGNSAGYG------IAKVVNSTEEAERVIESGDILVVKDADIDYVSILNRVS 54
E_NP_244029_BACHAL|584        EVVAKGQGIGRDTVNG------KVVIAANAEEANAKVEEGSILVTFSTDKDMMPAFEKAA 54
E_NP_693092_OCEIHE|586        DVIAKGQGIGRRSAYG------HAVFAKTNKEALEKVQDGDILVTYATEKEMMPAIEKAS 54
E_2E28_A|Geobacillus_stearothe DLLAKGQGIGRKSAFG------KAVVAKTAEEARQKMVDGGILVTVSTDADMMPAIEKAA 54
E_Q02499_KPYK_BACST|587       DLLAKGQGIGRKSAFG------KAVVAKTAEEARQKMVDGGILVTVSTDADMMPAIEKAA 54
E_P51181_KPYK_BACLI|585       DVVAKGQGIGRKSAFG------EVVIAQNAQEAAKKMKDGAVLVTKSTDRDMMASLEKAA 54
E_P80885_KPYK_BACSU|585       DIIAKGQGIGRKSAYG------PVVVAQNAKEAEQKMTDGAVLVTKSTDRDMIASLEKAS 54
E_P51182_KPYK_BACPY|586       DLLARGQGIGKDVAYG------RTVVAKNAAEALAYDTEGAILVTNASDRDMMPAIEKCA 54
E_3T05_A|Staphylococcus_aureus DEIANGQGIGRGSVVG------TTLVAETVKDLEGKDLSDKVIVTNSIDETFVPYVEKAL 54
E_NP_372221_STAAUR|585        DEIANGQGIGRGSVVG------TTLVAETVKDLEGKDLSDKVIVTNSIDETFVPYVEKAL 54
E_NP_764928_STAEPI|585        DEIAKGQGVGRGSVVG------HAIVADSASDLEGKDLSDKVIITNSVDETLVPYVEKAI 54
E_NP_834305_BACCER|584        EEIAKGQGIGRKAAKG------KVVVAKTAAEAVANVNEGDILVTTSTDKDMIPAIEKAA 54
E_NP_658626_BACANT|584        EEVAKGQGIGRKAAKG------KVVVAKTAAEAVANVNEGDILVTTSTDKDMIPAIEKAA 54
E_NP_470941_LISINN|585        EKVVKGQGIGSKSVIG------KAVVAKSNAEALQKAEEGGILIVKTTDKEILPAFEKSA 54
E_NP_465095_LISMON|585        EKVVKGQGIGSKSVIG------KAIVAKSNAEALEKAEEGGILIVKTTDKEILPAFEKSA 54
E_NP_785440_LACPLA|586        SKLADGQGVGDETVIG------KAVIATSAQEAIDKAVEGGVLVTKTTDKDYLPAIEKSS 54
E_AAP72039_LACCAS|587         QLVQ-GSGVGDESTIG------KAVIASNAQEAAAKMQKGDILVVKTTDKDYLPAIEKAA 53
E_NP_814779_ENTFAE|585        SKLVQGQGVGEEAIIA------KAVVAATAEEAVAKATEGAILVTKTTDKEYMPAIEKAS 54
E_P34038_KPYK_LACDE|589       NQLAQGLGVGTGSVIG------KAVVANSAEEANAKVHEGDILVAKTTDKDYMPAIKKAS 54
E_AAS08902_LACJOHN|589        TQLVKGLGVGEQSVIG------KAVVATSAEEANSKVQDGDILVAKTTDKDYLPAIKKAS 54
E_ZP_00046514_LACGASS|589     TQLVKGLGVGEQSVIG------KAVVATSAEEANSKVQDGDILVAKTTDKDYLPAIKKAS 54
K_NP_488048_NOSSP|589         AVLGQGIGLGQGLVSG-------CARVAHTGMDVGNFNAGDILVAPRTGADFVEAIRKAG 53
K_ZP_00106833_NOSPUNT|589     AVLGHGIGLGQGLVSG-------RARVANTGMDVSNFNPGDILVAPRTSADFVEAIRKSA 53
K_ZP_00070905_TRYERI|587      AVLGQGTGVGQGSVSG-------RARVARNSTEVRDFNHGEILVVNQTNAEFVEAMQKAS 53
K_NP_683065_THEELO|594        SVMGRGCGIGHGSVSG-------PARVVRQGMPVRTFNRGEILVAERTSAELVEVIRKAA 53
K_NP_926441_GLOVIO|605        AILGRGLGLGQNAVSG-------RARIVRHSDEAAALSPGDILIAPRTDANFIEAIKLAG 53
K_NP_440894_SYNCYS|591        AILGRGVGIGQGAVSG-------RARVASRPQAIAQFTQGEILVVPSTNADCVDMMRRAA 53
K_NP_894511_PROMAR|605        AVLGQGIGIGNGTVSG-------KVRFAFSPNDSTKIEPGEILVIHETSDDYLDAIRKSA 53
```

```
K_NP_893030_PROMAR|596          KVVTRGISVGEIGVTG-------KARNIKTNLDLSLISPGEILFVPKNILMDLPFTKIIT 53
NPA75450|Methanobacteriota_arc  EVLARGTGFGNSPVAGKVCRYPEICDITVIDSYPHNLNVSGAKALIIESKVYNPAVLRAL 60
E_D3T9H6_ACIB4|547              DVIARGKGYGKKKIRGKICRDAENCDILLMSHWNEI-NTENVRGIVIESKIYEPSKLINL 59
WP_012997271|Candidatus_Acidul  DVIARGKGYGKKKIRGKICRDAENCDILLMSHWNEI-NTENVRGIVIESKIYEPSKLINL 59
E_L0HNF6_ACIS0|545              EVIARGNGFGEKIVHGKICRIPRKCDIVLLSNIDSSEQPENVRGIVVESDTYDPSLLREI 60
WP_015283492|Aciduliprofundum_  EVIARGNGFGEKIVHGKICRIPRKCDIVLLSNIDSSEQPENVRGIVVESDTYDPSLLREI 60
HDD43544|Candidatus_Desulfofer  DILGKGVAFGKRRVRGKVCFGMNERGEILVRKEMPEIFTLLSFKGVIIENEIFNPEIIKK 60
MDL1956310|Candidatus_Desulfof  DILGKGVAFGKRRVRGKVCFGINERGEILVRKEMPEIFTLLSFKGVIIENEIFNPEIIKK 60
WP_276899524|Dictyoglomus_ther  DVIGQGHGLGGEAKTAK-------VIIAKNAKEAISKVTEDVILLVPTFEEELEIILDKL 53
WP_149122399|Dictyoglomus_ther  DVIGQGHGLGGEAKTAK-------VIIAKNAKEAISKVTEDVILLVPTFEEELEIILDKL 53
WP_012547033|Dictyoglomus_ther  DVIGQGHGLGGEAKTAK-------VIIAKNAKEAISKVTEDVILLVPTFEEELEIILDKL 53
WP_273334432|Dictyoglomus_turg  DVIGQGHGLGGEAKTAK-------VIIAKNAKEAISKVTEDVILLIPTFEKELEIILDKL 53
WP_012584142|Dictyoglomus_turg  DVIGQGHGLGGEAKTAK-------VIIAKNAKEAISKVTEDVILLIPTFEKELEIILDKL 53
WP_287019969|Dictyoglomus_sp.|  DVIGQGHGLGGEAKTAK-------VIIAKNAKEAISKVTEDVILLIPTFEKELEIILDKL 53
WP_011177137|Picrophilus_oshim  KFIGRGYPVGNSFSGYYEPDGSGDIIILDKYIKNFDYKKYKCVIIKGNVSDTIKNLFYEN 60
WP_084272625|Picrophilus_oshim  KFIGRGYPVGNSFSGYYEPDGSGDIIILDKYIKNFDYKKYKCVIIKGNVSDTIKNLFYEN 60
E_Q6L281_PICTO|555              KFIGRGYPVGNSFSGYYEPDGSGDIIILDKYIKNFDYKKYKCVIIKGNVSDTIKNLFYEN 60
MCL4329577|Thermoplasmatota_ar  NFVGRGYAFGRKVQGPCMEKDGDIVITGPQEETGRKNGIRAIITRNRLEYDIIERLAGER 60
E_KPYK_THEAC|544                KFIGRGYSFGDSLSGTATYGTKGDILMSEDGRIPGTDFRAFIFTSDIKPSLMSSLKGKTV 60
MCY0852041|Thermoplasma_acidop  KFIGRGYSFGDSLSGTATYGTKGDILMSEDGRIPGTDFRAFIFTSDIKPSLMSSLKGKTV 60
WP_010901306|Thermoplasma_acid  KFIGRGYSFGDSLSGTATYGTKGDILMSEDGRIPGTDFRAFIFTSDIKPSLMSSLKGKTV 60
WP_297025905|Thermoplasma_sp.|  KFIGRGYSFGESLSGTATYTGNGDILVADDGKTPNGDFKGYIFTSDIRPSLMSSIKGKTV 60
WP_297218145|Thermoplasma_sp.|  KFIGRGYSFGESVNGTATYNGNGDILVVEDGKIPNKNFKAFIFTVDPKPSVIDSLNGKTV 60
WP_237265313|Thermoplasma_sp._  KFIGRGYSFGDSINGTATYSGNGDILVVEDGKIPDKNFKAFIFTVDPKPSVIGSLNGKTI 60
PYB68239|Thermoplasma_sp._Kam2  KFIGRGYSFGDSINGTATYSGNGDILVVEDGKIPDKNFKAFIFTVDPKPSVIGSLNGKTI 60
WP_010917278|Thermoplasma_volc  EFLGRGYPQGKDAFGTATYGKNGNILISNSKDVPDGNFDAYIFTADVKPSEVKKINGKTA 60
E_Q979W1_THEVO|541              EFLGRGYPQGKDAFGTATYGKNGNILISNSKDVPDGNFDAYIFTADVKPSEVKKINGKTA 60
WP_276922945|Ferroplasma|559    RFMGRGYPAGTSVTGKVDTDVPENGNIILLDKYEGTIDYSKYDAIIIKSDVTNRIIDKFL 60
MEM4835156|Ferroplasma_sp._|35  RFMGRGYPAGTSVTGKVDTDVPENGNIILLDKYEGTIDYSKYDAIIIKSDVTNRIIDKFL 60
WP_021786504|Ferroplasma_sp._T  RFMGRGYPAGTSVTGKVDPDIPENGNIILLDKYENTIDYSKYKAIIIKSDVTNRIIDKFL 60
WP_337860350|Ferroplasma_sp.|5  RFIGRGYPAGSSLTGKVDPVHPENGNIIILDKYDDALDYSKYNAAIIKSNVTNRIIDKFV 60
MCL4332682|Thermoplasmatota_ar  SFLGRGYPAGPSLKGRVTMNTPGDGDIVLLTSLKDVKAVSGFSGIILQGRVNAGTADALR 60
WP_009886736|Ferroplasma|559    RFIGRGYPAGHNVTGKIDTDAPENGNIILLDRYDSNIDYSKYNAAIIKSDVTNRIIDKFL 60
E_S0APK2_FERAC|559              RFIGRGYPAGHNVTGKIDTDAPENGNIILLDRYDSNIDYSKYNAAIIKSDVTNRIIDKFL 60
WP_298279037|Ferroplasma_sp.|5  TFMGRGYPAGRSITGKIDIDRSGQGNIIILDRYDSSIDFSKYDAAIIKSDVTNRIIDTFI 60
WP_309215187|Ferroplasma_sp.|5  TFIGRGYPAGKSITGKIDIDRSGQGNIIILDRYDSSIDFSKYDAAIIKSDVTNRIIDTFM 60
MEM0139049|Ferroplasma_sp.|559  TFVGRGYPVGKSVSGTINYGGNGTGNIILLEEYDSSINFSKYDGIIIKSSVTNRIIDQFM 60
MCL4307372|Thermoplasmatota_ar  KFMGRGYPSGKNFKGTVTSKLDRSGDIYVTKNLRGARISDLADFTGIIFLEGIGHASEEE 60
MEM0157422|Thermoplasmataceae_  KFMGRGYPSGKNFKGTITSKLSKPGDIYVTNNLKGIRISDLAEYTGIIFLESISHASVEE 60
MCL4331222|Thermoplasmatota_ar  KFMGRGYPSGKNFKGTVTWKLDGPGDIYVTENLKGARISDLAEFTGIIFLESIGHASEEE 60
MFG1545118|Thermoplasmataceae_  KFMGRGYPSGKNFKGTVTWKLDGPGDIYVTENLKGARISDLAEFTGIIFLESIGHASEEE 60
EQB70352|Thermoplasmatales_arc  KFMGRGYPSGKNFKGTVTWKLDGPGDIYVTENMKGARISDLAEFAGIIFLESIGHASEEE 60
MEM0158203|Thermoplasmataceae_  SFVGRGYPSGKSIRGRLTRNPDEKREILLVEGQAQIPFEKIGKFQAVIFTNQISHRYAEN 60
MFG1449288|Thermoplasmataceae_  TFIGRGYPSGKSVKGKITRNPDEKREILLVESDEEISFDRIGKYQAIIFTGNVSNKMRET 60
MCL4438505|Thermoplasmatota_ar  TFIGRGYPSGKSVKGKITRNPDEKREILLVESDEDISFDRIGKYQAIIFTGNVSNKMRET 60
MCL4337896|Thermoplasmatota_ar  NFVGRGYPAGKSLTGKAFINKSGKGDILVLDTQEIPEGIKIEKLKAVIFIPNISTKLKES 60
MCL5782750|Thermoplasmatota_ar  NFMGRGYPYGKSVKGTVALKSGEPGDILICSRADLPENPDYGHLKAVIFTTYISHSLRES 60
MCL6091152|Thermoplasmatota_ar  NFMGRGYPYGKSVKGTVALKPGEPGDILICTRADLPENLDYGRLKAVIFTTYIPHSLRER 60
MCL4336005|Thermoplasmatota_ar  NFMGRGYPYGKSVKGTVALKSGEPGDILICTRADLPENLDYGRLKAVIFTTYIPHSLRER 60
MEM0155607|Thermoplasmataceae_  RYLGVGHANGISADGTVTYGTDGNCNILVVSKEDEIPLNAS-KYDGIICQSAVSSFLRER 59
WP_229657491|Thermogymnomonas_  RFIGRGYPCGKSTDGVVTRNIDGTGDVLITDSLSERDMDRYGKFRCVVCTSSVPHRLLRE 60
GGM73108|Thermogymnomonas_acid  RFIGRGYPCGKSTDGVVTRNIDGTGDVLITDSLSERDMDRYGKFRCVVCTSSVPHRLLRE 60
MCL4340783|Thermoplasmatota_ar  KFLGRGYPSGKSFRGKVAGKHKKGDILIVKDDISEEREDFMKYRGIIFTGKASHEVMDAL 60
WP_393971732|Oxyplasma_meridia  KFLGRGYPSGKSFKGIVAGEKNSGDILIVKDDISEEKKDFLKYKGIIFTGKASHEVMEAL 60
MCL5802698|Thermoplasmatota_ar  KFLGRGYPVGKSFRGKVAGNHLKGDILIVNDDVSEPRDEFKIYRGIIFTGKASHDVMDSL 60
MDP8012128|Thermoplasmata_arch  EFLGRGYPVGKSFSGKITKSGKNGNILLVNKRIIDVDKKFLKYKGIIFIEDVSPQIMDFL 60
MCL4420203|Thermoplasmatota_ar  NFIGRGYPLGKSVDGKVTLKPSGKGDILVASGNTVSESAESRFRGIIFTDDIPQKLKNEL 60
MCL5989634|Thermoplasmatota_ar  NFIGRGYPLGKSVDGKVTLKQSGKGDILVVSGNTVSESVESRFRGIIFTEDIPQKLKNEL 60
MCW6168190|Thermoplasmatales_a  NFVGRGYPLGKSINGKITLKQSGKGDILVVSGNTVNESAASRFRGIIFTEDIAQKLKNDL 60
WP_287954990|Acidiplasma_sp.|3  AFLGRGYAVGKSIKGKINTGNGSGNILIADSYDKNAEYTKFDAVIFRSCVSERIKDYFIN 60
WP_287961213|Acidiplasma_sp.|5  AFLGRGYAVGKSIKGKINTGNGSGNILIADSYDKNAEYTKFDAVIFRSCVSERIKDYFIN 60
WP_055041068|Acidiplasma|557    AFLGRGYAVGKSIKGKINTGNGSGNILIADSYDKNAEYTKFDAVIFRSCVSERIKDYFIN 60
WP_048100774|Acidiplasma|557    AFLGRGYAVGKSIKGKINTGNGSGNILIADSYDKNAEYTKFDAVIFRSCVSERIKDYFIN 60
MFG1519237|Thermoplasmataceae_  DYVGRGYATGKKVTGKYMPDGTGEIIVSERFSKDLLQRKGTKAMIIMERILADDIETISK 60
MCL4412357|Thermoplasmatota_ar  KFIGRGYSKGKTVEGRVTYNPDGGGEILVTDQGRLPDNYEFRAVIFENRPLPSTVEHLLE 60
MEM3534131|Thermoplasmatales_a  DFIGRGFATGPFCAGKATYGRDKKGDIFISSKPDDSIPQQSKGVLFTYRPPLYKVDSLVR 60
MEM3362986|Thermoplasmatales_a  DFIGRGFATGPFCAGKATYGRDKKGDIFISSKPDDSIPQQSKGVLFTYRPPLYKVDSLVR 60
MEM3313589|Thermoplasmatales_a  DFIGRGFATGPFCAGKATYGRDKKGDIFISSKPDDSIPQQSKGVLFTYRPPLYKVDSLVR 60
MEM3224589|Thermoplasmatales_a  DFIGRGFATGPFCAGKATYGRDKKGDIFISSKPDDSIPQQSKGVLFTYRPPLYKVDSLVR 60
MEM0312197|Thermoplasmatales_a  DFIGRGFATGPFCAGKATYGRDKKGDIFISSKPDDSIPQQSKGVLFTYRPPLYKVDSLVR 60
MEM1613726|Thermoplasmatales_a  DFIGRGFATGPFCAGKATYGRDKKGDIFISSKPDDSIPQQSKGVLFTYRPPLYKVDSLVR 60
MEM0073950|Thermoplasmatales_a  DFIGRGFATGPFCAGKATYGRDKKGDIFISSKPDDSIPQQSKGVLFTYRPPLYKVDSLVR 60
MEM3801201|Thermoplasmatales_a  DFIGRGFATGPFCAGKATYGRDKKGDIFISSKPDDSIPQQSKGVLFTYRPPLYKVDSLVR 60
MEM4090127|Thermoplasmatales_a  DFIGRGFAFGPFCAGKATYGRDKKGDIFISSKPDDSIPQQSKGVLFTYRPQLYKVDSLVR 60
MEM0128455|Thermoplasmatales_a  DFIGRGFAFGPFCAGKATYGRDKKGDIFISSKPDDSIPQQSKGVLFTYRPQLYKVDSLVR 60
MEM0141264|Thermoplasmatales_a  DFIGRGFAFGPFCAGKATYGRDKKGDIFISSKPDDSIPQQSKGVLFTYRPQLYKVDSLVR 60
MEM0138217|Thermoplasmatales_a  DFIGRGFAFGPFCAGKATYGRDKKGDIFISSKPDDGIPQQSKGVLFTYRPPLYKVDLLVR 60
MEM3496635|Thermoplasmatales_a  DFIGRGFATGPFCAGKATYGRDKKGDIFISSKPDDSIPQQSKGILFTYRPPLYKVDSLVR 60
MEM4055520|Thermoplasmatales_a  DFIGRGFATGPFCAGKATYGRDKKGDIFISSKPDDSIPQQSKGILFTYRPPLYKVDSLVR 60
MEM4095056|Thermoplasmatales_a  DFIGRGFATGPFCAGKATYGRDKKGDIFISSKPDDSIPQQSKGILFTYRPPLYKVDSLVR 60
MEM3205724|Thermoplasmatales_a  DFIGRGFATGPFCAGKATYGRDKKGDIFISSKPDDSIPQQSKGILFTYRPPLYKVDSLVR 60
MEM3212538|Thermoplasmatales_a  DFIGRGFATGPFCAGKATYGRDKKGDIFISSKPDDSIPQQSKGILFTYRPPLYKVDSLVR 60

                                         70        80        90       100       110       120
                                ....|....|....|....|....|....|....|....|....|....|....|....|
E_M0GLR0_HALL2|585              GIVDARAGMTGYPALVARELDIPMVSG-APLPKTIAQGATVTLHAERGIVYEGDVINHDR 112
E_D4GUB6_HALVD|585              GIVDARAGMTGYPALVARELDIPMVSG-APLPKTIAQGATVTLHAERGIVYEGDVINHDR 112
E_M0I6D0_9EURY|585              GIVDARAGMTGYPALVARELDIPMVSG-APLPKTIAQGATVTLHAERGIVYEGDVINHDR 112
```

```
E_M0FFY9_9EURY|585    GIVDARAGMTGYPALVARELDIPMVSG-APLPKTIAQGATVTLHAERGIVYEGDVINHDR 112
E_M0FY16_9EURY|585    GIVDARAGMTGYPALVARELDIPMVSG-APLPKTIAQGATVTLHAERGIVYEGDVINHDR 112
E_M0FS45_9EURY|585    GIVDARAGMTGYPALVARELDIPMVSG-APLPKTIAQGATVTLHAERGIVYEGDVINHDR 112
E_M0IYL0_9EURY|585    GIVDARAGMTGYPALVARELDIPMVSG-APLPKTIAQGATVTLHAERGIVYEGDVINHDR 112
E_M0IA90_9EURY|585    GIVDARAGMTGYPALVARELDIPMVSG-APLPKTIAQGATVTLHAERGIVYEGDVINHDR 112
E_M0HJT1_9EURY|586    GIVDARAGMTGYPALVARELDIP-MVSGAPLPKTIKQGATVTLHAERGIVYEGDVISHDR 112
E_M0GLR8_9EURY|586    GIVDARAGMTGYPALVARELDIPMVSG-APLPKTIKQGATVTLHAERGIVYEGDVISHDR 112
E_M0I4Q1_9EURY|585    GIIDARAGMTGYPALVARELDIPMISG-APLPKVVEEGALVTLHAERGIVYEGDVISYNR 112
E_M0IZU3_HALMT|585    GIIDARAGMTGYPALVARELDIPMISG-APLPKTIEEGASVTLHAERGIVYEGDVISYNR 112
E_I3R2N6_HALMT|607    GIIDARAGMTGYPALVARELDIPMISG-APLPKTIEEGASVTLHAERGIVYEGDVISYNR 112
E_M0GPJ1_9EURY|586    GIVDARAGMTGYPALVARELDIPMISG-APLPTTVSDGSLVTLHAERGIVYEGDVIGFDR 112
E_M0HUA5_9EURY|586    GIVDARAGMTGYPALVARELDIPMISG-APLPTTVSDGSLVTLHAERGIVYEGDVIGFDR 112
E_E4NRC8_HALBP|586    GIIDARPGMTGYPALVARELDLPMVSG-APLPESIEPGTELTLHAERGVVYEGNVLKHES 112
E_M0CVL5_9EURY|594    GIIDARPGMTGYPALVARELDLPMISG-APIPQSVAAGTEITLHAERGIVYEGNVIKHES 112
E_Q18JV3_HALWD|586    GIIDARPGMTGYPAVIARELDIPMISG-APLPPQISDGTAITIHAERGIVYEGDLTRSHD 112
E_G0LGR5_HALWC|586    GIIDARPGMTGYPAVIARELDIPMISG-APLPPQISDGTAITIHAERGIVYEGDLTRSHD 112
E_J3EZ11_9EURY|584    GIVDARAGMTGYPALVARELDIPMISG-APLPDELADGETVTLYAERGVVYEGDIIKHQQ 112
E_E7QTI2_9EURY|582    GIVDSRPGMTGYPALIARELEIPMISG-APLGPTVSDGDEITLDAERGVVYDGDVRSYDR 112
E_R4W8G0_9EURY|594    GIVDARKGMTGYPAMVARELEIPMISG-APMAESVAPGDEVTLDAERGVVYAGAIDPGAD 112
E_M0N190_9EURY|584    GIVSGHKGVTGYPAIVARELGIPMIGG-VDLA-DLPADVTVTLDAERGVVYEGDITSTR- 110
E_M0M4X2_HALMO|584    GIISGHKGVTGYPAIVARELGIPMIGG-VDLA-DLPEDVTVTLDAERGVVYEGDITSTR- 110
E_M0M9D2_9EURY|589    GIVSAHKGVTGYPAIVARELDLPMTSA-VALPDDLQDGTTVTLDAERGVVYEGDIPGLSG 112
E_M0M5X3_9EURY|581    GVVSAHQGVTGYAAIVARETGLPMISN-VSLPDSLADGDEVTLDAGRGVVYEGRVGN--- 109
E_M0MKB2_9EURY|580    GIVSAHEGVTGYPAIVARETGLPMISN-VSLP-GVDEGDSVTLDAGRGVVYEGSVGG--- 108
E_M0N0N7_9EURY|580    GIVSAHEGVTGYPAIVAREAGLPMVSN-VSLS-DVDEGDHVTLDAGRGVVYEGSVGG--- 108
E_F7PNS4_9EURY|582    GIIDAHTDRRGHAVTVGRRLDIPTASH-MTVPADIEDGATVTLDAERGVLYKGQLGTLDE 112
E_C7NP64_HALUD|582    GIIDGHTNRRGHAVTVGRRLDIPTVSH-VTVPDELEDGATVTLDAERGVLYRGQLGTLDD 112
E_C7NYS9_HALMD|581    GIVDAHDGTTGYAAIVARELSIPMIAN--AELPDLADGTQVTLDAERGIVYEDTTEDDRS 111
E_M0CQS7_9EURY|590    GVIDRHGGMTSYAAIVARELDVAMISN-ASLPREVESGTVVTLDAERGIVYDEAIQSRAR 112
E_G0HY69_HALHT|596    GIIDAHEGVTSYAAIVARELSIPMISN-ADLPDAVSDGSTVTLDSERGVVYEEAVGREDI 119
E_M0JGH6_HALVA|596    GIIDAHEGVTSYAAIVARELSIPMISN-ADLPDAVSDGSTVTLDSERGVVYEEAVGREDI 119
E_M0LC35_HALJP|596    GIIDAHEGVTSYAAIIARELSIPMISN-ADLPDTVSDGSTVTLDSERGVVYEEAVGREDI 119
E_M0KPP3_9EURY|596    GIIDAHEGVTSYAAIVARELSIPMISN-ADLPDAVSDGSTVTLDSERGVVYEEAVGREDI 119
E_M0KV41_HALAR|596    GIIDGHEGVTSYAAIVARELSIPMISN-ADLPDAVSDGSTVTLDSERGVVYEEAVGREDI 119
E_Q5V4I8_HALMA|610    GIIDGHEGVTSYAAIVARELSIPMISN-ADLPDAVSDGSTVTLDSERGVVYEESVGREDI 119
E_M0JZ82_9EURY|596    GIIDGHEGVTSYAAIVARELSIPMISN-ADLPDAVSDGSTVTLDSERGVIYEESVGREDI 119
E_M0K0G8_9EURY|610    GIIDGHEGVTSYAAIVARELSIPMISN-ADLPDAVSDGSTVTLDSERGVVYEESVGREDI 119
E_M0BVS9_9EURY|583    GIVDTRPGMTGYAALVARELGIPMLS--DADCTTVEAGRVVTIDGDRGVVYDGDVGTDGT 109
E_L0I815_HALRX|583    GIVDTRPGMTGYAALVARELGIPMLS--DADCTTVEAGRIVTIDGDRGVVYDGDVGTDGS 109
E_D8J7J0_HALJB|594    AIVDARSGMTGYPAMIARELGIPMVSG--ADLTEVPDGTTVTVDAERGVIYEGELASPTE 111
E_Q9HSA5_HALSA|579    AIVSADAGLTGYPAVIARELDVPMVG--DVDVDAVPAGDLVTVDGERGVVYEADQ----- 106
E_B0R347_HALS3|579    AIVSADAGLTGYPAVIARELDVPMVG--DVDVDAVPAGDLVTVDGERGVVYEADQ----- 106
E_G4IHY5_9EURY|577    GIVSAQSGMTGYPAMVARELDVPMVG--DVDVATVPANTLVTVDGERGVVYAADP----- 106
E_M0B9C3_9EURY|584    GIVNARRGMTGYPALVAREMDVPMVSG--ATISEMEAGTVVTIDAERGVVYEGDIRDRTD 111
E_L9ZS75_9EURY|584    GIVNARRGMTGYPALVAREMDVPMVSG--AAISEIEAGTVVTIDAERGVVYEGDIRDRTD 111
E_M0AKF4_NATA1|584    GIVNARRGMTGYPALVAREMDVPMVSG--ATISEMETGTVVTIDAERGVVYEGDIRDRTD 111
E_D3SYX2_NATMM|588    GIVNAQRGMTGYPALVAREINVPMVSG--ADIPDAASGTVVTVDAERGVVYEGNISDRRN 111
E_M0AAI6_9EURY|593    GIVNAQRGMTGYPALVAREIDVPMVSG--ADIPDTDAGTVVTVDAERGVVYEGDIGARHH 111
E_L9ZVH2_9EURY|593    GIVNAQRGMTGYPALVAREMDVPMVSG--ADIPDTDTGTVVTVDAERGVVYEGNIGDRRD 111
E_D2RXQ5_HALTV|585    GIIDAQRGLTGYPALVAREMDIPMISG--ADLEEAADGDVVTLDAERGVVYGGDLGERTD 111
E_M0BWI7_9EURY|585    GIIDAQRGLTGYPALIAREMDIPMISG--ADLEETVDGDVVTLDAERGVVYGGDLGARAD 111
E_L9WF02_9EURY|585    GIIDAQRGLTGYPALVAREMSIPMISG--AVLTAVENSDVVTLDAERGVVYGGDISDRAN 111
E_L9W4T0_9EURY|585    GIIDAQRGLTGYPALVAREMGIPMISG--ADPAGPADGDIVTLDAERGVVYGGDIGHRAD 111
E_L9W3Q0_9EURY|599    GIVDAQRGLTGYPALVAREMNIPMVSG--ADLSAAEEGSVVTLDAERGVIYGGDLSDRTD 111
E_L9XBF4_9EURY|585    GIVDAQRGLTGYPALVAREMDVPMISG--VDRTELVDGDVVTVDAERGVVYGGEIGTRTD 111
E_F8D381_HALXS|585    GIIDAQRGMTGYPSLVAREMDLPMISG--ADVSEEAEGETVTIDAERGVVYGGDIGDQAV 111
E_L9XI31_9EURY|586    GIVNAQRGMTGYPALVAREIDVPMISG--ADVDELEDGSTVTLDAERGVVYGGNIAPRSE 111
E_L0JWJ5_9EURY|586    GIVNAQRGMTGYPALVAREMDVPMISG--ADVDELEDGATVTLDAERGVVYGGSIGDRTE 111
E_M0MBJ8_9EURY|593    GILNAERGMTGYPALVAREMDLPMISD--AAVAETDVGTTVTLDAERGVVYRGEIADRTQ 111
E_M0L2S3_9EURY|605    GILNAQRGMTGYPALVAREMDIPMISD--AEVSETDVGTTVTLDAERGVVYRGEIGDRTE 111
E_L0ADH0_NATGS|585    GIVNAERGMTGYPALVAREMDVPMISD--AAVSELEDGTTVTVDAERGVVYAGEIGDRTN 111
E_M0BLU5_9EURY|585    GIVNAQRGMTGYPAMIARELGVPMISD--ADVAALSAGETVTVDAERGVVYGGDIGGRPE 111
E_L0JRU4_NATP1|585    GIVNAQRGMTGYPAMIARELGVPMISD--ADVETLSAGETVTVDAERGVVYGGDIGGRPE 111
E_M0CUB6_9EURY|585    GIVNAERGMTGYPALVARELGIPMLSD--ADVTALTEGEAVTLDAEHGVVYGGDIGDRHE 111
E_L9YBC2_9EURY|585    GIINAERGMTGYPALVARELEIPMISD--ADGTDIAAGEVVTVDAERGVVYGGDISDRTV 111
E_L9YIB5_9EURY|585    GIVNAERGMTGYPALVARELELPMISD--ADIADLEDGEIVTIDAERGIVYGGEIGDRTE 111
E_L9ZZY6_9EURY|585    GIVNAERGMTGYPALVARELELPMISD--AEIGDLEDGEIVTIDAERGIVYGGEIGDRTE 111
E_L9YUZ4_9EURY|585    GIINAERGMTGYPALVARELELPMISD--AEIADLEDGEIVTIDAERGIVYGGEIGDRTE 111
E_I7C1X6_NATSJ|585    GIINAERGMTGYPALVARELELPMISD--AEIADLEDGEIVTIDAERGIVYGGEIGDRTE 111
E_G2MMV7_9ARCH|599    GIVDAGTRMTSYAAIVAREMGVPIVVD-AELGSDVRDGDQITIDGHRGVVYDGSVERDHG 112
E_M0PIF3_9EURY|585    GIIDAREGMTGYPAVVARELGVPMVSG-AQLPENVGDGTVVTLDGERGIVYDGDVIAAAQ 112
E_M0NXB3_9EURY|585    GIIDAREGMTGYPAVVARELGVPMVSG-ATLPENVADGTVITLDGERGIVYDGDVIAAAR 112
E_B9LTN0_HALLT|585    GIVDAREGMTGYPAVVARELGVPMVSG-ARLPESVTDGTVVTVDGERGVVYDGDVIAATQ 112
E_M0DSX7_9EURY|585    GIVDAREGMTGYPAVIARELGVPMVSG-APLPESVTDGTVVTVDGERGVVYDGDVIAAAR 112
E_M0NZ95_9EURY|585    GIVDAREGMTGYPAVVARELGVPMVSG-ARLPESVTDGTVVTVDGERGVVYDGDVIAAAR 112
E_M0DB95_9EURY|585    GIVDAREGMTGYPAVVARELGVPMVSG-ARLPESVVDGTTVTVDGERGVVYDGDVIAAAR 112
E_M0FJ51_9EURY|585    SIVDAREGMTGYPAVVARELGVPMVSG-ARLPESVVDGTTVTVDGERGVVYDGDVIAAAR 112
E_M0EM72_9EURY|585    GIIDAREGMTGYPAVVARELGVPMVSG-AGLPESVADGTTVTVDGERGVVYDGDVLRAAR 112
E_M0EC36_9EURY|585    GIVDAREGMTGYPAVVARELGVPMVSG-AALPESVADGTTVTVDGERGVVYDGDVLRAAR 112
E_M0DGP4_9EURY|585    GVIDAREGMTGYPAVVARELGVPMVSG-AGLPESVADGTTVTVDGERGVVYDGDVLRAAR 112
E_M0PND9_9EURY|585    GVIDAREGMTGYPAVVARELGVPMVSG-AGLPESVADGTTVTVDGERGVVYDGDVLRAAR 112
E_M0NY16_9EURY|585    GVIDAREGMTGYPAVVARELGVPMVSG-AGLPESVADGTTVTVDGERGVVYDGDVLRAAR 112
E_M0F352_9EURY|585    GVIDAREGMTGYPAVVARELGVPMVSG-AGLPESVADGTTVTVDGERGVVYDGDVLRAAR 112
E_M0ER95_9EURY|585    GVIDAREGMTGYPAVVARELGVPMVSG-AGLPESVADGTTVTVDGERGVVYDGDVLRAAR 112
E_Q3ISC3_NATPD|593    GIVAVDEGLTGYPAIIARELDVPMVSG-ATVPAAVEEGADITVDGDRAVVYEGDVVSRRT 112
E_A7U0W4_9EURY|577    AVVAADRGMTSYAAMVARELGVPMVGG---VDVDATDGTVVTVDGDRGVVYESEVAER-- 108
K_Q5NW05_9ARCH|583    AIISEETGLTSYSAIIGRELNIPVVVGIKNATRIIQDGSTITVDSVRGMVYEGSINLPGE 113
E_M1XZK5_9EURY|580    AIVTAQSGMTSYPAMIARELGVPMVG---DATLSLSGGDVVTVDGDRGVVYEAAVDER-- 108
```

```
K_Q0W8N0_UNCMA|583               AIISEETGLTSYSAIIGRELNIPVVVGIKNATRIIQDGSTITVDSVRGMVYEGSINLPGE 113
K_H8I9P5_METCZ|583               AILSEESGLTSFSAIIGREMSIPVVVGIKDATTRFKDGMKVTIDTLSGLVYEGYINLPSD 113
K_D1YYS4_METPS|583               AILSEESGLTSFSAIIGREMNMPVVVGIKDATSKFNDGMKVTIDTVSGLVYEGFINLPGD 113
E_NP_623403_THETEN|583           AIITEEGGLTSHAAIVGLNLGIPVIVGCEGATSKLQDGMTVTVDTTRGLVYKGIVNIK-- 112
E_NP_783015_CLOTET|584           GVITERGGLTSHLAIECITNEIPFICGAGGATDVLKSGYFITMDVKRGIVYNGRITI--- 111
E_NP_244029_BACHAL|584           AVITEEGGITSHAAVVGLSLNTPVIVGVQNASALFKDGEEITVDTSRGDIYRGQASVL-- 112
E_NP_693092_OCEIHE|586           AIITEEGGLTSHAAVVGLSLGIPVIVGVDNVFEILQDGQDITVDGNRGDIYAGHAGVL-- 112
E_2E28_A|Geobacillus_stearothe   AIITEEGGLTSHAAVVGLSLGIPVIVGVENATTLFKDGQEITVDGGFGAVYRGHASVL-- 112
E_Q02499_KPYK_BACST|587          AIITEEGGLTSHAAVVGLSLGIPVIVGVENATTLFKDGQEITVDGGFGAVYRGHASVL-- 112
E_P51181_KPYK_BACLI|585          ALITEEGGLTSHAAVVGLSLGIPVIVGMENATSILKEGEDITVDSARGAVYKGRASVL-- 112
E_P80885_KPYK_BACSU|585          ALITEEGGLTSHAAVVGLSLGIPVIVGLENATSILTDGQDITVDASRGAVYQGRASVL-- 112
E_P51182_KPYK_BACPY|586          GLITEEGGLTSHGAIVGLSLGIPIIVGVENATELIQHGKEITMDAESGVIYNGHASVL-- 112
E_3T05_A|Staphylococcus_aureus   GLITEENGITSPSAIVGLEKGIPTVVGVEKAVKNISNNVLVTIDAAQGKIFEGYANVL-- 112
E_NP_372221_STAAUR|585           GLITEENGITSPSAIVGLEKGIPTVVGVEKAVKNISNNMLVTIDAAQGKIFEGYANVL-- 112
E_NP_764928_STAEPI|585           GLITEENGITSPSAIIGLEKGIPTVVGVEQATKEIKNDMLVTLDASQGKVFEGYANVL-- 112
E_NP_834305_BACCER|584           ALVVEEGGLTSHAAVVGVSIGIPVIVGVNGVTATLKNGQEVTVDAARGIVYNGABEL--- 111
E_NP_658626_BACANT|584           ALVVEEGGLTSHAAVVGVSIGIPVIVGVNGVTATLKNGQEVTVDAARGIVYNGABEL--- 111
E_NP_470941_LISINN|585           AVVVEEGGLTSHAAVVGINLGIPVIVGAKDATSLVKDGEIITVDSRQGVVYNGKTATH-- 112
E_NP_465095_LISMON|585           AVVVEEGGLTSHAAVVGINLGIPVIVGAKDATSLVKDGEIITVDSRQGVVYNGKTATH-- 112
E_NP_785440_LACPLA|586           ALVVENGGLTSHAAVVGISMGIPVIVGVKDATSVIADGQLITVDSRRGLVYRGASNAL-- 112
E_AAP72039_LACCAS|587            ALVVETGGLTSHAAVVGIAMGIPVVVGAENATSVISDGQIITVDSRRGIIYKGATNAL-- 111
E_NP_814779_ENTFAE|585           ALVVEEGGLTSHAAVVAIAQNIPVIVGAADATSLINNDEVITVDPRRGIVYRGATTAI-- 112
E_P34038_KPYK_LACDE|589          GMIVEASGLTSHAAVVGVSLGIPVVVGVADATSKIADGSTLTVDARRGAIYQGEVSNL-- 112
E_AAS08902_LACJOHN|589           GMVVEASGLTSHAAVVGLSLGIPVIVGATDAVEKIKDGSKITVDARRGAVYQGEATNL-- 112
E_ZP_00046514_LACGASS|589        GMVVEASGLTSHAAVVGLSLGIPVVVGATDAVEKIKDGSKITVDARRGAVYQGEATNL-- 112
K_NP_488048_NOSSP|589            GIITEDESLTSHAAVIGLRLGVPVIVGVKKATQVIRDGAILTLDLQRGLVYSGAVGTP-- 111
K_ZP_00106833_NOSPUNT|589        GIITEEESLTSHAAVIGLRLGVPVIVGVKQATQVIRDGAIITLDLQRGLIYSGAVRTP-- 111
K_ZP_00070905_TRYERI|587         GVVTEEDSVTSHAAVIGLRLGLPVIVGVKNATNVVRDGTILTLDAQRGMVYSGTNRR--- 110
K_NP_683065_THEELO|594           GIITEEDSLTSHAAVLGLRLGIPVIVGVKNATRLIREGTILTMDVERGVVYSGARNGGER 113
K_NP_926441_GLOVIO|605           GIVTEESGLTSHAAIIGLRLGLPVLLGVKDCTTRIPDGVVLTLDPQKGVIFSGAASAV-- 111
K_NP_440894_SYNCYS|591           GIITEEESLTSHAAIIGLRLGVPVIVGFKGATQKIRDGAIVTIDAQKGLIYSGALPPVSK 113
K_NP_894511_PROMAR|605           GVITEQEGEEYHAEMISKRLGIPFIIGIAHATRNLLEGEVVTMQLSNGKVHRGTGGKLPM 113
K_NP_893030_PROMAR|596           GIVTDENIDECYKVFKKNNIKISTICNLSAIDNLVSNGDLITLQLNEGVVYMGQIEDDED 113
NPA75450|Methanobacteriota_arc   ANRGVVALAGTGKIHKLPEELTVDPVRGVAWR---------------------------- 92
E_D3T9H6_ACIB4|547               SKKGISILAGTGKIEIENEVVTLDPERGVLWK---------------------------- 91
WP_012997271|Candidatus_Acidul   SKKGISILAGTGKIEIENEVVTLDPERGVLWK---------------------------- 91
E_L0HNF6_ACIS0|545               SRRGISILAGTGTLNVENEEVTLDPKRGIIWK---------------------------- 92
WP_015283492|Aciduliprofundum_   SRRGISILAGTGTLNVENEEVTLDPKRGIIWK---------------------------- 92
HDD43544|Candidatus_Desulfofer   AVKKGVTIIIGVKNILKHLKPGDVIQIDPKRGIVFR------------------------ 96
MDL1956310|Candidatus_Desulfof   AVKKGVTIIIGVKNILKHLKPGDVIQIDPKRGIVFR------------------------ 96
WP_276899524|Dictyoglomus_ther   QGIIIEDEKIPSTLIAWRSKGISIIVGAKGLSEKLKDNEIITIDPERGVIYRGVARLY-- 111
WP_149122399|Dictyoglomus_ther   QGIIIEDEKIPSTLIAWRSKGISIIVGAKGLSEKLKDNEIITIDPERGVIYRGVARLY-- 111
WP_012547033|Dictyoglomus_ther   QGIIIEDEKIPSTLIAWRSKGISIIVGAKGLSEKLKDNEIITIDPERGVIYRGVARVY-- 111
WP_273334432|Dictyoglomus_turg   QGIIIEDEKIPPFLIPWRSKGISIIVGAKNLSEKLKDNEIITIDPERGVIYRGVARIY-- 111
WP_012584142|Dictyoglomus_turg   QGIIIEDEKIPPFLIPWRSKGISIIVGAKNLSEKLKDNEIITIDPERGVIYRGVARIY-- 111
WP_287019969|Dictyoglomus_sp.    QGIIIEDEKIPPFLIPWRSKGISIIVGAKNLSEKLKDNEIITIDPERGVIYRGVARIY-- 111
WP_011177137|Picrophilus_oshim   KILLLYNTEFDAELNEKTFLNIDGDTGIITA----------------------------- 91
WP_084272625|Picrophilus_oshim   KILLLYNTEFDVELNEKTFLNIDGDTGIITA----------------------------- 91
E_Q6L281_PICTO|555               KILLLYNTEFDAELNEKTFLNIDGDTGIITA----------------------------- 91
MCL4329577|Thermoplasmatota_ar   TTVVHRTRFFRTPQNGETLLIDPGTGVIVSSSVKKVSK---------------------- 98
E_KPYK_THEAC|544                 VTKARLVRQIKEGERIYIDGNTGIILMASPDQK--------------------------- 93
MCY0852041|Thermoplasma_acidop   VTKARLVRQIKEGERIYIDGNTGIILMASPDQK--------------------------- 93
WP_010901306|Thermoplasma_acid   VTKARLVRQIKEGERIYIDGNTGIILMASPDQK--------------------------- 93
WP_297025905|Thermoplasma_sp.|   VTKARLVRRIQEGERIYIDGNTGIILTASINAKKNSSR---------------------- 98
WP_297218145|Thermoplasma_sp.|   VTKTRLVRKILEGERIFVDGNTGIILTSSQR----------------------------- 91
WP_237265313|Thermoplasma_sp._   VTKTRLVRKILEGEHIYIDGNTGIILTSSQR----------------------------- 91
PYB68239|Thermoplasma_sp._Kam2   VTKTRLVRKILEGEHIYIDGNTGIILTSSQR----------------------------- 91
WP_010917278|Thermoplasma_volc   VFKTRLARTINEGERIYIDGNTGIILKYEK------------------------------ 90
E_Q979W1_THEVO|541               VFKTRLARTINEGERIYIDGNTGIILKYEK------------------------------ 90
WP_276922945|Ferroplasma|559     DLGKTLVYNAELVEKIDKNITISIDADTGIITGI-------------------------- 94
MEM4835156|Ferroplasma_sp._|35   DLGKTLVYNAELVEKIDKNITISIDADTGIITGI-------------------------- 94
WP_021786504|Ferroplasma_sp._T   DLGKTLVYNAELVEKIDKNITISIDADTGIITGI-------------------------- 94
WP_337860350|Ferroplasma_sp.|5   DSGKTLVYSAEIVEKIDKITSISIDADTGIITAI-------------------------- 94
MCL4332682|Thermoplasmatota_ar   DSGKTVLFSARFIQLPGEGDEIYIDAETGVVLGSGKPGDQNRSSRTD------------- 107
WP_009886736|Ferroplasma|559     DAGKTLVYNAELVKQINKNTTISIDADTGIITEI-------------------------- 94
E_S0APK2_FERAC|559               DAGKTLVYNAELVKQINKNTTISIDADTGIITEI-------------------------- 94
WP_298279037|Ferroplasma_sp.|5   QAGKTLVYNTEFVSEIKTGSTISIDADTGIITCI-------------------------- 94
WP_309215187|Ferroplasma_sp.|5   QAGKTLVYNTELVSEIKTGSTISIDADTGIITCI-------------------------- 94
MEM0139049|Ferroplasma_sp.|559   RLGKTLVYGTEITSKIDSNKKINIDGDTGIITYIN------------------------- 95
MCL4307372|Thermoplasmatota_ar   LSETGIPFIHNTKLLKGLDDGTNVYVDGSTGVITS------------------------- 95
MEM0157422|Thermoplasmataceae_   LSETGIPFIHNTKLLKELDDGINVYVDGSTGVITS------------------------- 95
MCL4331222|Thermoplasmatota_ar   LSETGIPFIHNTKLLKGLEDGTNVYVDGSTGVITS------------------------- 95
MFG1545118|Thermoplasmataceae_   LSETGIPFIHNTKLLKGLEDGTNVYVDGSTGVITS------------------------- 95
EQB70352|Thermoplasmatales_arc   LSETGIPFIHNTKLLKSLEDGTNVYVDGSTGVITS------------------------- 95
MEM0158203|Thermoplasmataceae_   LRGMGITILTNTKLASYVRDGEEVYLDGETGVLIL------------------------- 95
MFG1449288|Thermoplasmataceae_   LRDVGITVLHNTRLAVYIKDGEDVFVDGETGVIIH------------------------- 95
MCL4438505|Thermoplasmatota_ar   LRDVGITVLHNTRLAVYIKDGEDVFVDGETGVIIH------------------------- 95
MCL4337896|Thermoplasmatota_ar   IREKGVTVLSNTRFSIIIKPGDTLHIDGCTGVITQ------------------------- 95
MCL5782750|Thermoplasmatota_ar   IKEKGLTVVHNTKLVRELVTGETIHIDGATGVIIS------------------------- 95
MCL6091152|Thermoplasmatota_ar   IKERGLTVVHNTKLVRELKAGETIHIDGATGVIIS------------------------- 95
MCL4336005|Thermoplasmatota_ar   IKERGLTVVHNTKLVRELKAGETIHIDGATGVIIS------------------------- 95
MEM0155607|Thermoplasmataceae_   LRESETSLLSNAKLFYDIKEGDNVFIDGYTGIVVS------------------------- 94
WP_229657491|Thermogymnomonas_   LTSRGVTVLSNTRLAGDVKEGDHVTVDGDTGVING------------------------- 95
GGM73108|Thermogymnomonas_acid   LTSRGVTVLSNTRLAGDVKEGDHVTVDGDTGVING------------------------- 95
MCL4340783|Thermoplasmatota_ar   ETSGIPVLYNTKIREKISEGDEIFIDGYTGIINS-------------------------- 94
WP_393971732|Oxyplasma_meridia   ESSGIPVLYNTKIREKISAGDEIFIDGYTGIINS-------------------------- 94
MCL5802698|Thermoplasmatota_ar   NAMGISVLYNTKIKEKISEGDQIFVDGYTGIINS-------------------------- 94
MDP8012128|Thermoplasmata_arch   ETNKISALYNTKLYKVPEEGEIVYIDGYTGTMIR-------------------------- 94
```

```
MCL4420203|Thermoplasmatota_ar  ISKKITILYRTRLAGGIDEGQMVHIDGFTGVIIS-------------------------- 94
MCL5989634|Thermoplasmatota_ar  ISKKITILYRTRLAGGIDEGQMVHIDGFTGVIIS-------------------------- 94
MCW6168190|Thermoplasmatales_a  ISKKTTILYRTRLAGGIDEGQMVHIDGFTGVIIS-------------------------- 94
WP_287954990|Acidiplasma_sp.|3  NNITLVYNTEFSSNIMPDMEINVDSDTGIITYI--------------------------- 93
WP_287961213|Acidiplasma_sp.|5  NNITLVYNTEFSSNIMPDMEINVDSDTGIITYI--------------------------- 93
WP_055041068|Acidiplasma|557    NNITLVYNTEFSSNIIPDMEINVDSDTGIITYI--------------------------- 93
WP_048100774|Acidiplasma|557    NNITLVYNTEFSSNIMPDMEINVDSDTGIITYI--------------------------- 93
MFG1519237|Thermoplasmataceae_  TGITVVQSARFVRDLKDEEEIMIDPGTGVIVSNSRSEEVPPE------------------ 102
MCL4412357|Thermoplasmatota_ar  KVNCVLVSTIFKNKPAEGEKVYIEARTGIIYQ---------------------------- 92
MEM3534131|Thermoplasmatales_a  DGKTVVSSVIFRRSPNEGEFLNIDPHTGIIYA---------------------------- 92
MEM3362986|Thermoplasmatales_a  DGKTVVSSVIFRRSPNEGEFLNIDPHTGIIYA---------------------------- 92
MEM3313589|Thermoplasmatales_a  DGKTVVSSVIFRRSPNEGEFLNIDPHTGIIYA---------------------------- 92
MEM3224589|Thermoplasmatales_a  DGKTVVSSVIFRRSPNEGEFLNIDPHTGIIYA---------------------------- 92
MEM0312197|Thermoplasmatales_a  DGKTVVSSVIFRRSPNEGEFLNIDPHTGIIYA---------------------------- 92
MEM1613726|Thermoplasmatales_a  DGKTVVSSVIFRRSPNEGEFLNIDPHTGIIYA---------------------------- 92
MEM0073950|Thermoplasmatales_a  DGKTVVSSVIFRRSPNEGEFLNIDPHTGIIYA---------------------------- 92
MEM3801201|Thermoplasmatales_a  DGKTVVSSVIFRRSPNEGEFLNIDPHTGIIYA---------------------------- 92
MEM4090127|Thermoplasmatales_a  DGKTVVSSVIFRRSPDEGEFLNIDPHTGIIYA---------------------------- 92
MEM0128455|Thermoplasmatales_a  DGKTVVSSVIFRRSPDEGEFLNIDPHTGIIYA---------------------------- 92
MEM0141264|Thermoplasmatales_a  DGKTVVSSVIFRRSPDEGEFLNIDPHTGIIYA---------------------------- 92
MEM0138217|Thermoplasmatales_a  DGKTVVSSVIFRRSPNEGEFLNIDPHTGIIYA---------------------------- 92
MEM3496635|Thermoplasmatales_a  DGKTVVSSVIFRRSPNEGEFLNIDPHTGIIYA---------------------------- 92
MEM4055520|Thermoplasmatales_a  DGKTVVSSVIFRRSPNEGEFLNIDPHTGIIYA---------------------------- 92
MEM4095056|Thermoplasmatales_a  DGKTVVSSVIFRRSPNEGEFLNIDPHTGIIYA---------------------------- 92
MEM3205724|Thermoplasmatales_a  DGKTVVSSVIFRRSPNEGEFLNIDPHTGIIYA---------------------------- 92
MEM3212538|Thermoplasmatales_a  DGKTVVSSVIFRRSPNEGEFLNIDPHTGIIYA---------------------------- 92

                                        130
                                ....|....|....
E_M0GLR0_HALL2|585              DSR----------  115
E_D4GUB6_HALVD|585              DSR----------  115
E_M0I6D0_9EURY|585              DSR----------  115
E_M0FFY9_9EURY|585              DSR----------  115
E_M0FY16_9EURY|585              DSR----------  115
E_M0FS45_9EURY|585              DSR----------  115
E_M0IYL0_9EURY|585              DSR----------  115
E_M0IA90_9EURY|585              DSR----------  115
E_M0HJT1_9EURY|586              GNRQ---------  116
E_M0GLR8_9EURY|586              GNRK---------  116
E_M0I4Q1_9EURY|585              GSR----------  115
E_M0IZU3_HALMT|585              GSR----------  115
E_I3R2N6_HALMT|607              GSR----------  115
E_M0GPJ1_9EURY|586              SERD---------  116
E_M0HUA5_9EURY|586              SERD---------  116
E_E4NRC8_HALBP|586              TRDP---------  116
E_M0CVL5_9EURY|594              DRTKAAKERPGN-  124
E_Q18JV3_HALWD|586              QDRN---------  116
E_G0LGR5_HALWC|586              QDRN---------  116
E_J3EZ11_9EURY|584              QR-----------  114
E_E7QTI2_9EURY|582              S------------  113
E_R4W8G0_9EURY|594              REGRSLDSAERV-  124
E_M0N190_9EURY|584              -------------  110
E_M0M4X2_HALMO|584              -------------  110
E_M0M9D2_9EURY|589              -------------  112
E_M0M5X3_9EURY|581              -------------  109
E_M0MKB2_9EURY|580              -------------  108
E_M0N0N7_9EURY|580              -------------  108
E_F7PNS4_9EURY|582              -------------  112
E_C7NP64_HALUD|582              -------------  112
E_C7NYS9_HALMD|581              -------------  111
E_M0CQS7_9EURY|590              ERRAGRDI-----  120
E_G0HY69_HALHT|596              SGRERDY------  126
E_M0JGH6_HALVA|596              SGSERDY------  126
E_M0LC35_HALJP|596              SGRERDY------  126
E_M0KPP3_9EURY|596              SGRERDY------  126
E_M0KV41_HALAR|596              SGRERDY------  126
E_Q5V4I8_HALMA|610              SGRERDY------  126
E_M0JZ82_9EURY|596              SGRERDY------  126
E_M0K0G8_9EURY|610              SGRERDY------  126
E_M0BVS9_9EURY|583              ER-----------  111
E_L0I815_HALRX|583              ER-----------  111
E_D8J7J0_HALJB|594              RREIDAPDWAGGR  124
E_Q9HSA5_HALSA|579              -------------  106
E_B0R347_HALS3|579              -------------  106
E_G4IHY5_9EURY|577              -------------  106
E_M0B9C3_9EURY|584              R------------  112
E_L9ZS75_9EURY|584              R------------  112
E_M0AKF4_NATA1|584              R------------  112
E_D3SYX2_NATMM|588              QDE----------  114
E_M0AAI6_9EURY|593              RDDQPSRHTQ---  121
E_L9ZVH2_9EURY|593              RDARSSRYTQ---  121
E_D2RXQ5_HALTV|585              RP-----------  113
E_M0BWI7_9EURY|585              RP-----------  113
E_L9WF02_9EURY|585              RT-----------  113
E_L9W4T0_9EURY|585              RR-----------  113
E_L9W3Q0_9EURY|599              RDEQVELA-----  119
E_L9XBF4_9EURY|585              RD-----------  113
E_F8D381_HALXS|585              RE-----------  113
```

```
E_L9XI31_9EURY|586              RN-----------  113
E_L0JWJ5_9EURY|586              RN-----------  113
E_M0MBJ8_9EURY|593              RV-----------  113
E_M0L2S3_9EURY|605              RT-----------  113
E_L0ADH0_NATGS|585              RV-----------  113
E_M0BLU5_9EURY|585              RP-----------  113
E_L0JRU4_NATP1|585              RP-----------  113
E_M0CUB6_9EURY|585              RA-----------  113
E_L9YBC2_9EURY|585              RR-----------  113
E_L9YIB5_9EURY|585              RL-----------  113
E_L9ZZY6_9EURY|585              RL-----------  113
E_L9YUZ4_9EURY|585              RL-----------  113
E_I7C1X6_NATSJ|585              RL-----------  113
E_G2MMV7_9ARCH|599              SVSSNGE------  119
E_M0PIF3_9EURY|585              QR-----------  114
E_M0NXB3_9EURY|585              ER-----------  114
E_B9LTN0_HALLT|585              KR-----------  114
E_M0DSX7_9EURY|585              KR-----------  114
E_M0NZ95_9EURY|585              QR-----------  114
E_M0DB95_9EURY|585              ER-----------  114
E_M0FJ51_9EURY|585              ER-----------  114
E_M0EM72_9EURY|585              ER-----------  114
E_M0EC36_9EURY|585              ER-----------  114
E_M0DGP4_9EURY|585              ER-----------  114
E_M0PND9_9EURY|585              ER-----------  114
E_M0NY16_9EURY|585              ER-----------  114
E_M0F352_9EURY|585              ER-----------  114
E_M0ER95_9EURY|585              ER-----------  114
E_Q3ISC3_NATPD|593              NGTETSVRDR---  122
E_A7U0W4_9EURY|577              -------------  108
K_Q5NW05_9ARCH|583              -------------  113
E_M1XZK5_9EURY|580              -------------  108
K_Q0W8N0_UNCMA|583              -------------  113
K_H8I9P5_METCZ|583              -------------  113
K_D1YYS4_METPS|583              -------------  113
E_NP_623403_THETEN|583          -------------  112
E_NP_783015_CLOTET|584          -------------  111
E_NP_244029_BACHAL|584          -------------  112
E_NP_693092_OCEIHE|586          -------------  112
E_2E28_A|Geobacillus_stearothe  -------------  112
E_Q02499_KPYK_BACST|587         -------------  112
E_P51181_KPYK_BACLI|585         -------------  112
E_P80885_KPYK_BACSU|585         -------------  112
E_P51182_KPYK_BACPY|586         -------------  112
E_3T05_A|Staphylococcus_aureus  -------------  112
E_NP_372221_STAAUR|585          -------------  112
E_NP_764928_STAEPI|585          -------------  112
E_NP_834305_BACCER|584          -------------  111
E_NP_658626_BACANT|584          -------------  111
E_NP_470941_LISINN|585          -------------  112
E_NP_465095_LISMON|585          -------------  112
E_NP_785440_LACPLA|586          -------------  112
E_AAP72039_LACCAS|587           -------------  111
E_NP_814779_ENTFAE|585          -------------  112
E_P34038_KPYK_LACDE|589         -------------  112
E_AAS08902_LACJOHN|589          -------------  112
E_ZP_00046514_LACGASS|589       -------------  112
K_NP_488048_NOSSP|589           -------------  111
K_ZP_00106833_NOSPUNT|589       -------------  111
K_ZP_00070905_TRYERI|587        -------------  110
K_NP_683065_THEELO|594          SPE----------  116
K_NP_926441_GLOVIO|605          -------------  111
K_NP_440894_SYNCYS|591          G------------  114
K_NP_894511_PROMAR|605          KLDSMD-------  119
K_NP_893030_PROMAR|596          ALDKYKYV-----  121
NPA75450|Methanobacteriota_arc  -------------  92
E_D3T9H6_ACIB4|547              -------------  91
WP_012997271|Candidatus_Acidul  -------------- 91
E_L0HNF6_ACIS0|545              -------------  92
WP_015283492|Aciduliprofundum_  -------------  92
HDD43544|Candidatus_Desulfofer  -------------  96
MDL1956310|Candidatus_Desulfof  -------------  96
WP_276899524|Dictyoglomus_ther  -------------  111
WP_149122399|Dictyoglomus_ther  -------------  111
WP_012547033|Dictyoglomus_ther  -------------  111
WP_273334432|Dictyoglomus_turg  -------------  111
WP_012584142|Dictyoglomus_turg  -------------  111
WP_287019969|Dictyoglomus_sp.|  -------------  111
WP_011177137|Picrophilus_oshim  -------------  91
WP_084272625|Picrophilus_oshim  -------------  91
E_Q6L281_PICTO|555              -------------  91
MCL4329577|Thermoplasmatota_ar  -------------  98
E_KPYK_THEAC|544                -------------  93
MCY0852041|Thermoplasma_acidop  -------------  93
WP_010901306|Thermoplasma_acid  -------------  93
WP_297025905|Thermoplasma_sp.|  -------------  98
WP_297218145|Thermoplasma_sp.|  -------------  91
WP_237265313|Thermoplasma_sp._  -------------  91
```

```
PYB68239|Thermoplasma_sp._Kam2  -------------  91
WP_010917278|Thermoplasma_volc  -------------  90
E_Q979W1_THEVO|541              -------------  90
WP_276922945|Ferroplasma|559    -------------  94
MEM4835156|Ferroplasma_sp._|35  -------------  94
WP_021786504|Ferroplasma_sp._T  -------------  94
WP_337860350|Ferroplasma_sp.|5  -------------  94
MCL4332682|Thermoplasmatota_ar  -------------  107
WP_009886736|Ferroplasma|559    -------------  94
E_S0APK2_FERAC|559              -------------  94
WP_298279037|Ferroplasma_sp.|5  -------------  94
WP_309215187|Ferroplasma_sp.|5  -------------  94
MEM0139049|Ferroplasma_sp.|559  -------------  95
MCL4307372|Thermoplasmatota_ar  -------------  95
MEM0157422|Thermoplasmataceae_  -------------  95
MCL4331222|Thermoplasmatota_ar  -------------  95
MFG1545118|Thermoplasmataceae_  -------------  95
EQB70352|Thermoplasmatales_arc  -------------  95
MEM0158203|Thermoplasmataceae_  -------------  95
MFG1449288|Thermoplasmataceae_  -------------  95
MCL4438505|Thermoplasmatota_ar  -------------  95
MCL4337896|Thermoplasmatota_ar  -------------  95
MCL5782750|Thermoplasmatota_ar  -------------  95
MCL6091152|Thermoplasmatota_ar  -------------  95
MCL4336005|Thermoplasmatota_ar  -------------  95
MEM0155607|Thermoplasmataceae_  -------------  94
WP_229657491|Thermogymnomonas_  -------------  95
GGM73108|Thermogymnomonas_acid  -------------  95
MCL4340783|Thermoplasmatota_ar  -------------  94
WP_393971732|Oxyplasma_meridia  -------------  94
MCL5802698|Thermoplasmatota_ar  -------------  94
MDP8012128|Thermoplasmata_arch  -------------  94
MCL4420203|Thermoplasmatota_ar  -------------  94
MCL5989634|Thermoplasmatota_ar  -------------  94
MCW6168190|Thermoplasmatales_a  -------------  94
WP_287954990|Acidiplasma_sp.|3  -------------  93
WP_287961213|Acidiplasma_sp.|5  -------------  93
WP_055041068|Acidiplasma|557    -------------  93
WP_048100774|Acidiplasma|557    -------------  93
MFG1519237|Thermoplasmataceae_  -------------  102
MCL4412357|Thermoplasmatota_ar  -------------  92
MEM3534131|Thermoplasmatales_a  -------------  92
MEM3362986|Thermoplasmatales_a  -------------  92
MEM3313589|Thermoplasmatales_a  -------------  92
MEM3224589|Thermoplasmatales_a  -------------  92
MEM0312197|Thermoplasmatales_a  -------------  92
MEM1613726|Thermoplasmatales_a  -------------  92
MEM0073950|Thermoplasmatales_a  -------------  92
MEM3801201|Thermoplasmatales_a  -------------  92
MEM4090127|Thermoplasmatales_a  -------------  92
MEM0128455|Thermoplasmatales_a  -------------  92
MEM0141264|Thermoplasmatales_a  -------------  92
MEM0138217|Thermoplasmatales_a  -------------  92
MEM3496635|Thermoplasmatales_a  -------------  92
MEM4055520|Thermoplasmatales_a  -------------  92
MEM4095056|Thermoplasmatales_a  -------------  92
MEM3205724|Thermoplasmatales_a  -------------  92
MEM3212538|Thermoplasmatales_a  -------------  92
```
